# Trends in Incidence of Atrial Fibrillation–Related Stroke: The Norwegian AFNOR Study

**DOI:** 10.1161/JAHA.126.049696

**Published:** 2026-09-09

**Authors:** Mariam Anjum, Inger Ariansen, Vidar Hjellvik, Jannicke Igland, Lars Jøran Kjerpeseth, Elisabeth Kvalvaag, Håkon Ihle‐Hansen, Arnljot Tveit, Ingrid Christophersen, Hanne Ellekjær, Marius Myrstad, Trygve Berge

**Affiliations:** ^1^ Department of Medical Research Bærum Hospital, Vestre Viken Hospital Trust Gjettum Norway; ^2^ Faculty of Medicine Institute of Clinical Medicine, University of Oslo Oslo Norway; ^3^ Department of Chronic Diseases Norwegian Institute of Public Health Oslo Norway; ^4^ Department of Global Public Health and Primary Care University of Bergen Norway; ^5^ Department of Neurology Haukeland University Hospital Bergen Norway; ^6^ Department of Medical Genetics Oslo University Hospital Oslo Norway; ^7^ Department of Neuroscience Norwegian University of Science and Technology Trondheim Norway; ^8^ Stroke Unit, Department of Internal Medicine St. Olav's Hospital Trondheim Norway

**Keywords:** atrial fibrillation, intracerebral hemorrhage, oral anticoagulation, risk factors, stroke, Atrial Fibrillation, Epidemiology, Risk Factors, Secondary Prevention, Ischemic Stroke

## Abstract

**Background:**

The increasing prevalence of atrial fibrillation (AF), along with changes in cardiovascular risk factors, AF detection and oral anticoagulant use, may have altered the epidemiology of AF‐related stroke. However, population‐based data on long‐term trends remain limited.

**Methods:**

Using mandatory nationwide registry data from the Atrial Fibrillation in Norway (AFNOR) study (2001–2021), we identified incident stroke cases defined as first hospitalization or death due to stroke in individuals aged ≥15 years. Age‐standardized incidence rates were calculated using direct standardization. Sex‐ and age‐adjusted incidence rate ratios (IRR) were estimated using negative binomial regression.

**Results:**

Among 227 575 incident strokes, 66 795 (29.4%) were AF‐related, and increased from 22% to 31% during 2001–2021, with 69% of all AF‐related ischemic strokes occurring in non‐anticoagulated individuals. Incidence rate of AF‐related ischemic strokes remained stable overall (67.8 per 100 000 person‐years in 2001 to 68.4 in 2021; IRR=1.01; 95% CI, 1.00–1.03) but increased among individuals aged 15 to 64 years. Non‐AF ischemic strokes declined from 204.4 to 130.8 (IRR=0.98; 95% CI, 0.97–0.99). Incidence of AF‐related intracerebral hemorrhage in individuals aged ≥75 years increased, driven by anticoagulated patients. Incidence of non‐AF intracerebral hemorrhage decreased (IRR=0.98; 95% CI, 0.97–0.99).

**Conclusions:**

The favorable decline in ischemic stroke incidence from 2001 to 2021 did not include AF‐related strokes. AF‐related ischemic strokes increased in younger age groups, whereas AF‐related intracerebral hemorrhage increased in the oldest age groups driven by anticoagulated patients. These findings underscore the need for improved strategies for AF detection and stroke prevention, particularly in younger populations, and greater attention towards management of bleeding risk among older, anticoagulated individuals.

## Graphical abstract

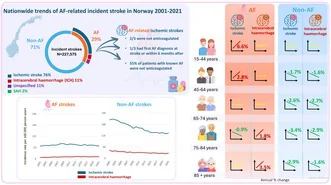

**Nonstandard Abbreviations and Acronyms**
ICH: intracerebral hemorrhage
IRR: incidence rate ratio
OAC: oral anticoagulation
SAH: subarachnoid hemorrhage

**Clinical Perspective**

**What Is New?**

- In this nationwide population‐based Norwegian study from 2001 to 2021, the incidence of atrial fibrillation ‐related stroke remained stable overall and increased in younger individuals, whereas non‐atrial fibrillation‐related stroke declined.
- The incidence of atrial fibrillation‐related intracerebral hemorrhage increased in older individuals, largely driven by oral anticoagulant users.

**What Are the Clinical Implications?**

- The findings highlight the need for improved stroke prevention specifically in younger patients with atrial fibrillation and underscore the importance of individualized risk–benefit assessment and management of bleeding risk in elderly patients.

Atrial fibrillation (AF) increases the risk of stroke 5‐fold compared with the general population, and AF‐related strokes are often more severe than other types of stroke, imposing a major public health challenge and economic burden.^1^ A meta‐analysis showed that 1 in 5 incident strokes were AF‐related, increasing to 1 in 3 in recent studies.^2^ Moreover, the increase in incidence and prevalence of AF itself is expected to continue over the next decades, raising concerns of a potential secondary increase in AF‐related strokes.^3^, ^4^

There is a lack of updated assessment of current temporal trends in AF‐related stroke incidence, and existing studies have shown conflicting results. Some studies have reported declining stroke rates related to increased oral anticoagulant (OAC) uptake, while recent studies have suggested stable trends, possibly due to the OAC uptake leveling off.^5^, ^6^, ^7^

Norwegian health registries are of high quality and nationwide data have been collected for decades. Norwegian registry data have shown stable incidence rates of AF during 2004–2014 among all age groups, except for an increase in incidence rates of AF in the population aged <45 years.^3^

While stroke incidence has declined over the last couple of decades in Norway, AF incidence has been stable and AF prevalence has increased, likely due to an aging population and improved detection,^3^, ^8^, ^9^ which raises concerns of a potential increase in AF‐related strokes. Hence, examining temporal trends in AF‐related strokes is important to optimize stroke prevention and management. Comorbidity burden is a prognostic factor for mortality and stroke risk, and with an aging population, monitoring important comorbidity trends is also of importance.^10^

This study aimed to examine temporal trends in the incidence of AF‐related ischemic stroke and intracerebral hemorrhage (ICH) in Norway from 2001 to 2021, and characterize potential differences across sex and age. Secondary aims included assessing trends in total stroke and other stroke subtypes. We also sought to explore how differences in comorbidity, including temporal patterns of CHA_2_DS_2_‐VA risk factors, and OAC use changed over the study period, and how these changes differed between AF‐related and non‐AF‐related ischemic strokes and ICH.

## METHODS

The study used nationwide registry data from the AFNOR (Atrial Fibrillation in Norway) study, linking individual‐level data from multiple population‐based registries, including the Norwegian Patient Registry, the Norwegian Cause of Death Registry, the Norwegian Prescription Database, the historical cardiovascular disease hospitalization database at the University of Bergen, and the Norwegian Control and Payment of Health Reimbursements Database covering primary health care. The historical cardiovascular disease database at the University of Bergen (1994–2007) and the Norwegian Patient Registry (2008–2021), were used to identify primary and secondary hospital discharge diagnoses codes of stroke according to the *International Classification of Diseases, Ninth Revision and Tenth Revision* (*ICD‐9* and *ICD‐10*). Details on the methodology, period of data availability, diagnoses and reimbursement codes used for the definition of AF, stroke, and comorbidities can be found in the Supplemental Material (Data S1 through S3).

The study was approved by the Norwegian Regional Committee for medical and Health Research Ethics, Health Region South‐East (REK 2020/82710). The requirement for informed consent was waived by the Committee.

The study data are available from the Norwegian Health Data Authority and Statistics Norway but are not publicly available because of licensing and data protection restrictions. Data and analytic methods may be made available from the authors upon reasonable request and approval from Norwegian Health Data Authority.

### Study Design and Study Population

The study was conducted as an open nationwide population‐based cohort study, including the entire Norwegian population aged ≥15 years between 2001 and 2021 (approximately 5.4 million). The population at risk was defined as the Norwegian population aged ≥15 years who had not been hospitalized with stroke or stroke sequela within the last 7 years. Person‐time at risk was censored upon death or emigration. Stroke incidence rates were calculated for each calendar year, with the number of incident stroke cases as the numerator and the person‐time of the population at risk as the denominator. Results were stratified by AF status, stroke subtype (ischemic, unspecified, intracerebral hemorrhage, subarachnoid hemorrhage), sex and age groups (15–44, 45–64, 65–74, 75–84 and 85+ years).

### Definition of Outcomes

An event of ischemic stroke, intracerebral hemorrhage (ICH), unspecified stroke or subarachnoid hemorrhage (SAH) was defined as a hospitalization with stroke as main or secondary diagnosis, or out‐of‐hospital death with stroke as underlying cause. Total stroke was defined as any of the above‐mentioned events. Stroke as a secondary diagnosis was not included if the primary diagnosis was previous stroke (*ICD‐9*: 438; *ICD‐10*: I69) or stroke rehabilitation (*ICD‐9*: V57; *ICD‐10*: Z50.80, Z50.89).

### Definition of AF, Comorbid Conditions, and OAC Treatment

Stroke was defined as AF‐related if AF was registered at any time during the period of data availability before the stroke, or within 6 months after stroke. Baseline comorbidities were defined using diagnosis codes registered until the stroke event, with a minimum look‐back period of 7 years. Diagnosis codes from primary care (from 2006), prescription reimbursement in the Norwegian Prescription Database (from 2008), inpatient contacts (from 1994) and outpatient contacts (from 2008) in the Norwegian Patient Registry, and the Norwegian Cause of Death Registry (from 2001) were used to define AF and comorbidities. Hence, AF and comorbidities were defined with minimum look‐back of 7 years. The CHA_2_DS_2_‐VA score was calculated at the time of stroke based on age, diagnosis codes and prescription claims.

OAC therapy at the time of incident stroke was assessed using prescription registry data. Individuals who claimed at least one prescription for any OAC within 6 months before the stroke event were defined as OAC users. The 6‐month window was chosen to capture regular use within 3 months and another 3‐month grace period to account for variations in prescription refills and patient adherence. OAC use was coded as a binary variable.

### Statistical Analysis

Categorical variables are presented as frequencies with percentages and continuous variables as means. The follow‐up time and calendar years were divided into 3 periods: 2001 to 2007, 2008 to 2012, and 2013 to 2021, to display changes in characteristics during follow‐up.

Age‐standardized annual incidence rates per 100 000 person‐years were calculated using direct standardization to the European Standard Population defined in 2013.^11^ Crude annual incidence rates were calculated by dividing the number of events by total person‐time in the population‐at‐risk and 95% CIs were calculated using the Poisson exact CI method. Age‐ and sex‐adjusted incidence rate ratios (IRR) were calculated using negative binomial regression analyses with calendar year as a continuous covariate, expressed as average annual percentage change in incidence rates. Results were stratified by AF‐status, sex and age group. Joinpoint regression was performed as a supplementary analysis to assess potential non‐linear trends in incidence rated of AF‐related and non‐AF related ischemic stroke and ICH. To assess whether temporal trends differed for sex or age group, interaction terms between calendar year and sex, and between calendar year and age group, were included. The significance of interaction was assessed using Wald test.

Incidence of AF‐related ischemic stroke was also analyzed according to timing of the first AF diagnosis in relation to stroke (AF diagnosed before stroke, diagnosed at the time of stroke, or within 6 months after stroke), with analysis restricted to 2010–2021 due to data availability. For AF‐related ischemic stroke and ICH, we additionally report the annual proportion with CHA_2_DS_2_‐VA comorbidities and OAC use by sex and age. As prescription data first became available in 2004, the OAC analyses were restricted to the period 2004–2021. Comorbidity reimbursement codes (diagnosis codes linked to conditions for which medication costs are reimbursed), were introduced in 2008, therefore analyses involving comorbidities were restricted from 2008 to 2021. To assess the impact of a longer and broader AF look‐back (more available registers in 2021 than in 2001), sensitivity analyses were conducted using a fixed look‐back period of 7 years for AF confined to the latter half of the period when all registries were available (2015–2021). Statistical analyses were performed using StataMP (version 18) and Joinpoint Regression Program (version 6.0.1.).

## RESULTS

### Proportion of AF

During 2001 to 2021, a total of 227 575 incident stroke cases were identified in Norway, of which 66 795 (29.4%) were AF‐related. Descriptive characteristics of stroke cases during the early, middle, and last part of the study period are shown in Table. Individuals with AF were older at the time of stroke, although the mean age declined slightly during the study period in both AF‐ and non‐AF‐related incident strokes. The CHA_2_DS_2_‐VA score was consistently higher among individuals with AF, and CHA_2_DS_2_‐VA comorbidities increased across the study period. The proportion of AF‐related strokes increased from 22.0% in 2001 to 31.8% in 2021 (Table S1). Of all AF‐related strokes, 51 059 (76.4%) were ischemic strokes, 7503 (11.2%) hemorrhagic strokes, 7182 (10.8%) were unspecified strokes and 1051 (1.6%) were SAH (Table S2). Among all AF‐related strokes, 70.6% (65.9% for ischemic strokes and 88.1% for ICH) occurred in individuals with previously known AF, i.e. AF was recorded at least once before the stroke date in the registers. AF was diagnosed at the time of stroke in 14.6% of AF‐related strokes, and within 6 months after stroke in 14.8%.

**Table 1:** Characteristics of the Stroke Population

	Non‐AF			AF		
	2001–2007	2008–2014	2015–2021	2001–2007	2008–2014	2015–2021
No.	61 516	52 071	47 193	20 385	23 354	23 056
Females, %	31 808 (51.7)	26 047 (50.0)	22 707 (48.1)	10 938 (53.7)	11 682 (50.0)	10 547 (45.8)
Mean age, SD	73.8 (14.1)	72.3 (14.9)	71.6 (14.8)	80.8 (8.7)	80.5 (10.1)	79.5 (10.7)
Median CHA_2_DS_2_‐VA score, IQR		3 (1–4)	3 (1–4)		4 (3–5)	4 (3–5)
Age <65 y	14 955 (24.3)	15 120 (29.0)	13 801 (29.2)	1173 (5.8)	1835 (7.9)	2131 (9.2)
Age 65–74 y	12 262 (19.9)	11 560 (22.2)	12 147 (25.7)	3102 (15.2)	4104 (17.6)	4808 (20.9)
Age >75 y	34 299 (55.8)	25 391 (48.8)	21 245 (45.0)	16 110 (79.0)	17 415 (74.6)	16 117 (69.9)
CHA_2_DS_2_‐VA score ≤2		24 306 (46.7)	21 341 (45.2)		4323 (18.5)	4270 (18.5)
CHA_2_DS_2_‐VA score >2		27 765 (53.3)	25 852 (54.8)		19 031 (81.5)	18 786 (81.5)
Heart failure		7220 (13.9)	6764 (14.3)		10 354 (44.3)	9764 (42.4)
Hypertension		31 489 (60.5)	31 101 (65.9)		17 680 (75.7)	18 890 (81.9)
Diabetes		7722 (14.9)	7996 (16.9)		4399 (18.8)	4722 (20.5)
Stroke, TIA, thromboembolism		10 875 (20.9)	10 454 (22.2)		6373 (27.3)	6991 (30.3)
Vascular disease		11 895 (22.8)	11 991 (25.4)		9464 (40.5)	9934 (43.0)
OAC use		1691 (3.3)	1861 (3.9)		7595 (32.5)	9654 (41.9)

Characteristics at time of stroke diagnosis stratified by 3 intervals. Age represents mean age at stroke. Values are n (%), mean (SD) or median (IQR). For 2001–2007, data on comorbidities and OAC were inconsistently recorded and are therefore not reported. IQR indicates interquartile range; OAC, oral anticoagulation; and TIA, transient ischemic attack.

### Incidence of Ischemic Stroke

From 2001 to 2021, the overall age‐standardized incidence rates remained stable for AF‐related ischemic strokes (IRR 1.01; 95% CI, 1.00–1.03) (Figure 1). Joinpoint analysis showed an initial increase until 2009 (average annual percentage change 1.99; 95% CI, 1.61–3.28), followed by a decline from 2009 to 2021 (average annual percentage change −1.08%; 95% CI, −1.64 to −0.65), resulting in a non‐significant trend over the full study period. Sex‐stratified incidence rates showed a similar biphasic trend, although men had numerically higher annual incidence rates than women (Table S3). No significant interaction between calendar year and sex was found (*P*‐interaction=0.924). Figure 2 illustrates the age‐stratified incidence rates of ischemic stroke during the study period. Temporal trends differed significantly across age groups (*P* for interaction <0.001). We found an increasing trend in AF‐related ischemic strokes in the age groups 15 to 44 and 45 to 64 years in both women and men. In women aged 65 to 74 years, joinpoint analysis found a trend shift in 2012 with increasing rates until 2012 and a declining trend thereafter. In contrast, incidence rates remained stable in men aged 65 to 74 throughout the study period, resulting in overall stable rates for both sexes combined. In individuals aged 75 to 84 years, a linearly decreasing trend was found in women, whereas an increase was observed in men until 2013 followed by a significant decline, resulting in overall stable rates for men and an overall declining trend for both sexes combined. In the age group ≥85 years, women had a declining trend with incidence rates that increased until 2008 before declining, whereas incidence rates remained stable in men due to only a transient decline during 2010–2013 with no significant change thereafter. Overall, incidence rates remained stable for both sexes combined (Figure S1).

**Figure 1:**
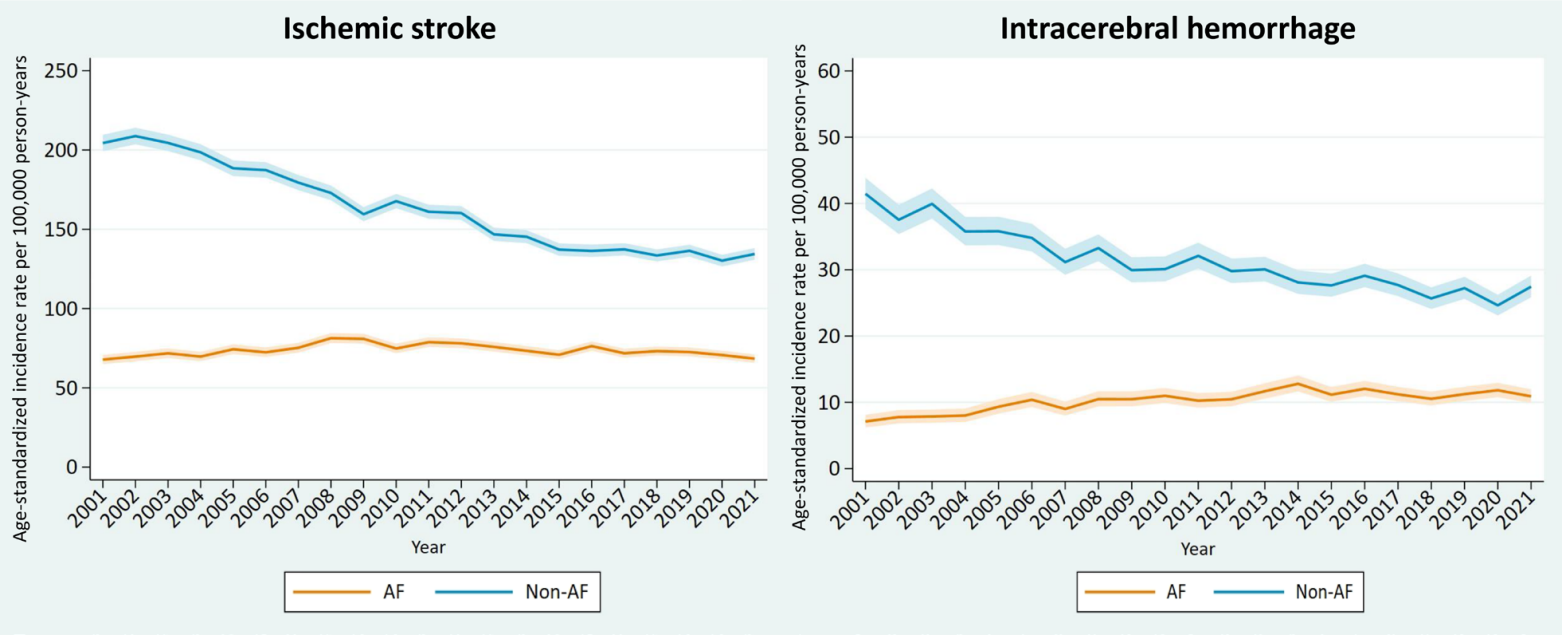
Incidence rates for ischemic stroke and intracerebral hemorrhage, stratified by atrial fibrillation. Age‐standardized annual incidence rates per 100 000 person‐years for ischemic stroke (left) and intracerebral hemorrhage (right), stratified by atrial fibrillation. The shaded area represents the 95% CI. Y‐axis scales differ between panels. AF indicates atrial fibrillation.

**Figure 2:**
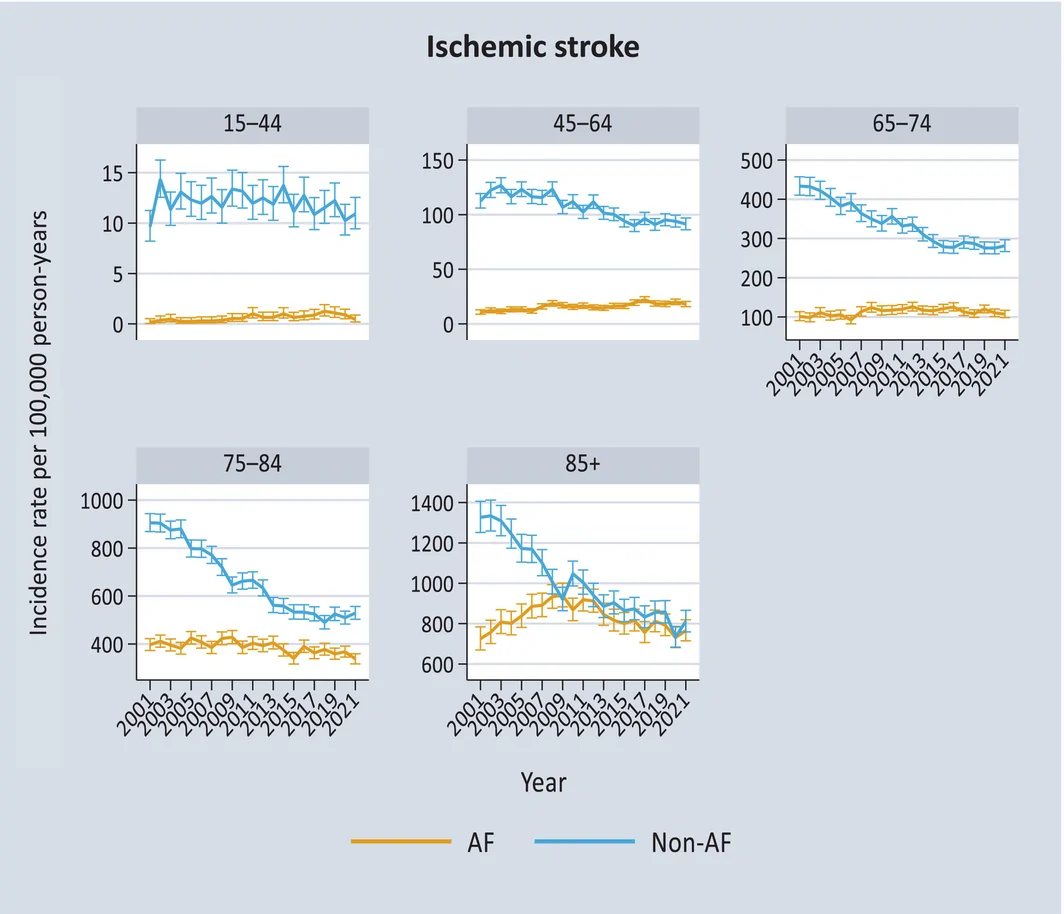
Incidence rates for AF‐related and non‐AF‐related ischemic stroke, stratified by age group. Incidence rates are presented with 95% CI. Y‐axis scales differ between panels. AF indicates atrial fibrillation.

Stratification by timing of AF diagnosis showed that the incidence rate of ischemic stroke in patients with known AF decreased from 55.88 (95% CI, 53.30–58.54) to 45.58 (95% 43.45–47.79) per 100 000 person‐years during 2010–2021, driven by individuals ≥75 years. The incidence remained stable for ischemic stroke presenting with AF at stroke, whereas the incidence rate for ischemic stroke with AF diagnosed within 6 months post‐stroke increased slightly from 14.64 (95% CI, 13.32–16.06) to 17.41 (95% CI, 16.11–18.80) over the same period (Figures S2 and S3). In comparison, non‐AF‐related ischemic strokes decreased over the study period (IRR 0.98; 95% CI, 0.97%–0.99%). The interaction between age groups and calendar year was also significant for non‐AF‐related ischemic strokes (*P*‐interaction=0.013), with no significant interaction between calendar year and sex (*P*‐interaction=0.924). The incidence rates were stable in the youngest age group (15–44 years; IRR=1.00; 95% CI, 0.99–1.00) but decreased significantly across all other age groups and in both sexes. Additional joinpoint analysis supported a declining trend.

### Incidence of ICH

Incidence rates of AF‐related ICH remained relatively stable with no statistically significant trend, increasing from 7.1 in 2001 to 11.1 in 2021 (IRR=1.01; 95% CI, 1.00–1.02). Joinpoint analysis indicated a significant increase until 2014 (average annual percentage change 3.54%; 95% CI, 2.47–7.48) and no significant change thereafter. This pattern was driven by men, whereas women exhibited a consistent linear increase. The interaction between sex and calendar year was not significant (*P*‐interaction=0.408), while the interaction between age group and calendar year was significant (*P* interaction=0.002). Age‐stratified analyses showed that the incidence of ICH increased significantly in individuals with AF aged 75 to 84 and above 85 years, with an IRR of 1.02 and 1.04, respectively, whereas the incidence was stable in the younger age groups (Figure 3). In the age group 65 to 74 years, joinpoint analysis showed an initial increase followed by a declining trend in men, whereas incidence rates remained stable in women throughout the study period, resulting in stable incidence rates for both sexes. Sex‐stratified analysis showed no differences. Stratifying the AF‐related ICH incidence by OAC use showed that the increase in AF‐related ICH incidence rates was driven mainly by anticoagulated individuals, whereas the ICH incidence rates declined in non‐anticoagulated individuals (Figure 4; Figure S4).

**Figure 3:**
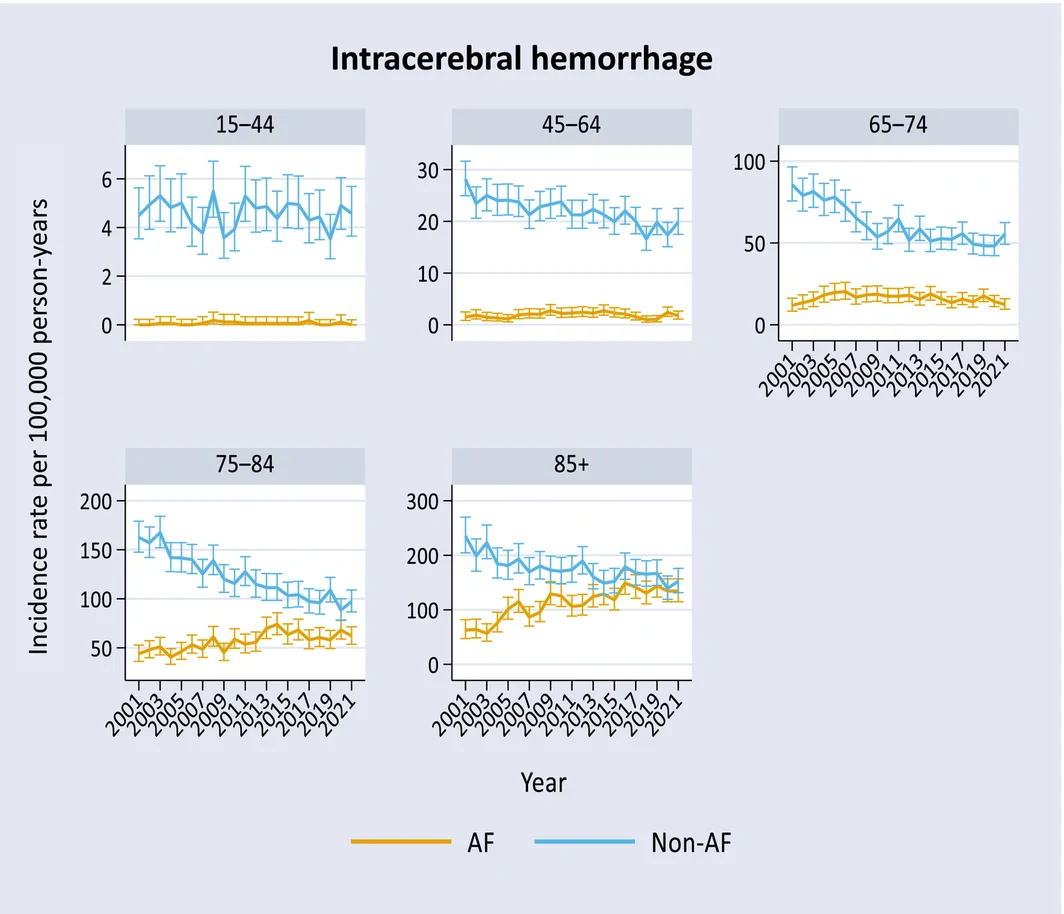
Incidence rate of AF‐related and non‐AF‐related intracerebral hemorrhage, stratified by age groups. Incidence rates are presented with 95% CI. Y‐axis scales differ between panels. AF indicates atrial fibrillation.

**Figure 4:**
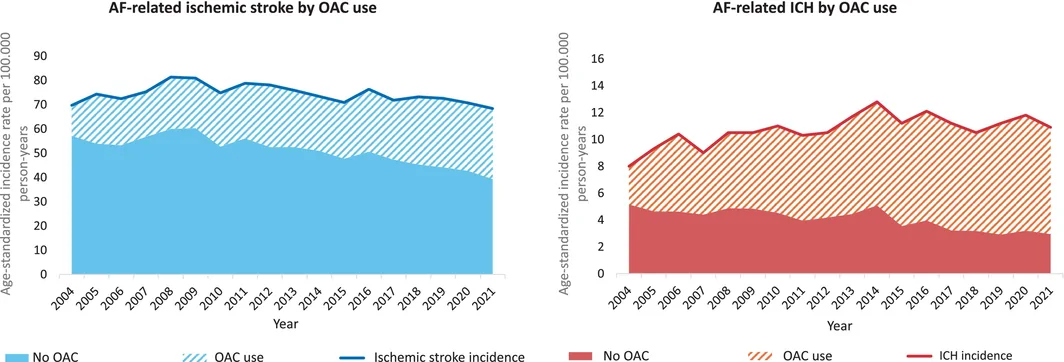
Age‐standardized incidence of AF‐related ischemic stroke and intracerebral hemorrhage with and without oral anticoagulation. The *left panel* shows the incidence of AF–related ischemic stroke per 100 000 person‐years. The dark blue line on top depicts the age‐standardized incidence rate of AF‐related ischemic stroke. The light blue area indicates subset of cases not receiving OAC, while the striped area represents the subset of cases using OAC. The *right panel* shows the incidence of AF–related intracerebral hemorrhage; the red line on top depicts the incidence of AF‐related ICH, the striped area represents the subset of cases using OAC while the solid red shaded area represents the subset of cases not using OAC. Note that the Y‐axis scales differ between the 2 panels. AF indicates atrial fibrillation; ICH, intracerebral hemorrhage; and OAC, oral anticoagulation.

Incidence rates of non‐AF ICH decreased during the study period (IRR=0.98; 95% CI, 0.97–0.99), with a consistent linear decline in all age groups, except for men and women aged 15 to 44, where the trends remained stable (Table S4). Temporal trends differed by age group (*P*‐interaction=0.004), with no significant interaction between calendar year and sex (*P*‐interaction=0.543).

### Subarachnoid Hemorrhage

There was an increase in AF‐related SAH (IRR 1.04; 95% CI, 1.03–1.05), corresponding to an average annual change of 4%, with similar trends for women and men (Table S5; Figure S5). Subgroup analyses showed a significant increase in all age groups, except those aged 15 to 44 years, who displayed an increasing but not statistically significant trend (IRR 1.05; 0.94–1.19). Stratification by both sex and age was not applied due to an insufficient sample size.

For non‐AF individuals, the overall incidence of SAH declined by 1.8% per year (IRR 0.98; 95% CI, 0.97–0.99). The declining trend was observed in all age groups, except in individuals aged ≥75 years, who showed a stable trend. The trends were similar for women and men.

### Unspecified Strokes

The proportion of AF‐related strokes classified as unspecified decreased from 12.2% in 2001 to 4.0% in 2021. The proportion of non‐AF unspecified strokes decreased from 16.9% in 2001 to 4.3% 2021. The incidence rate of unspecified stroke per 100 000 person years decreased from 10.8 in 2001 to 3.2 in 2021 (IRR 0.94; 95% CI, 0.93–0.95) in individuals with AF and from 47.1 to 7.5 in individuals without AF (IRR 0.91; 95% CI, 0.91–92) (Figure S6). A declining trend was observed in all age groups for both AF‐ and non‐AF‐related unspecified strokes, although the trend was not statistically significant for the youngest age group 15 to 44 years.

### Total Stroke

AF‐related total stroke incidence per 100 000 person‐years increased from 80.2 in 2001 to 101.3 in 2009 and thereafter declined gradually to 68.3 (95% CI, 65.6–71.0) by 2021 (Table S6; Figure S7). There was no significant overall trend (IRR 1.00; 0.99–1.02). Women had lower rates than men, but the trends overall were similar. AF‐related total strokes increased in those aged 15–44 years (IRR 1.06; 95% CI, 1.03–1.09) and 45 to 64 years (IRR 1.02; 95% CI, 1.02–1.03) in both sexes, was stable in those aged 65 to 74 years, declined in women aged 75 to 84 years but was stable in men, and decreased significantly in individuals aged ≥85 years (Figures S8 and S9).

Non‐AF strokes declined steadily during the study period from an incidence of 312.9 in 2001 to 182.4 in 2021 (IRR 0.97: 0.96–0.98). The decline was observed in both sexes and in all age groups. Age‐ and sex‐stratified incidence rates displayed similar trends, although men had numerically higher annual rates than women.

### Oral Anticoagulant Use

Among individuals with AF‐related ischemic stroke, 31.5% used OAC before the ischemic stroke, and the proportion of OAC users increased from 26.3% in 2008 to 42.6% in 2021. Age‐stratified analyses showed low OAC use among younger individuals, with higher uptake in older age groups. Similar patterns were observed in both men and women (Figure S10). Analyses across strata of CHA_2_DS_2_‐VA score, showed that among high‐risk individuals (CHA_2_DS_2_‐VA score ≥2) with AF‐related ischemic stroke, the proportion using OAC increased from 27.1% in 2008 to 45.7% in 2021 (Table S7).

In individuals with known AF before incident ischemic stroke, the proportion using OAC was 40.1% overall, and increased from 35.7% in 2008 to 62.3% in 2021. In high‐risk individuals with previously known AF, OAC use increased from 40.0% in 2008 to 64.2% in 2021 (Figure S11). Interestingly, among individuals with CHA_2_DS_2_‐VA score of 1, the proportion of OAC users with known AF before the incident ischemic stroke were relatively stable but decreased during the last 3 years of the study period from 37.8% in 2019 to 19.4% in 2021.

By contrast, 62.5% of all AF‐related ICH occurred in anticoagulated individuals. Among AF‐related ICH with known AF before incident ICH, OAC use increased from 62.6% in 2008 to 77.1% in 2021.

### Time Trends in the Distribution of Comorbidity in the Incident Stroke Population

Hypertension was the most common comorbidity in AF‐related ischemic stroke, followed by heart failure, vascular disease and diabetes (Figure 5). Among patients with AF with ischemic stroke, the proportion with hypertension increased steadily from 75.2% in 2009 to 82.3% in 2021, while heart failure decreased from 44.0% to 39.8%. Vascular disease increased slightly from 40.9% to 42.1%, while diabetes remained relatively stable. Age‐stratified analyses of AF‐related incident ischemic stroke showed that hypertension was the most common comorbidity across all age groups and increased gradually over time among those aged ≥65 years age (Figure S12). Heart failure and vascular disease decreased over time in patients with AF aged 45 to 64 years, while vascular disease increased in all other age groups. Diabetes remained largely stable. Men had higher prevalence of vascular disease in all age groups, with otherwise comparable comorbidity patterns between sexes.

**Figure 5:**
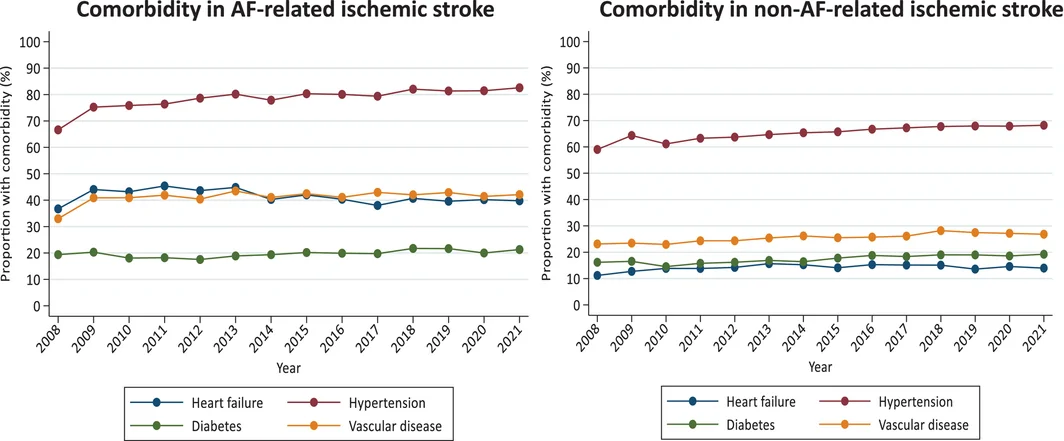
Annual proportion of comorbidities in AF‐related and non‐AF‐related ischemic stroke. AF indicates atrial fibrillation.

In non‐AF ischemic strokes, hypertension increased from 64.3% in 2009 to 68.2% in 2021. Heart failure and vascular disease prevalence was much lower in the non‐AF ischemic stroke population but increased slightly during the study period, whereas the proportion with diabetes increased from 16.5% in 2009 to 19.2% in 2021, reaching levels comparable to those with AF. Non‐AF ischemic stroke showed generally lower comorbidity levels than AF‐related ischemic strokes across all age groups, except for hypertension which was comparable in older age groups. By contrast, heart failure and vascular disease were consistently less prevalent in non‐AF individuals, with more pronounced differences in older age groups.

For ICH, comorbidity patterns were broadly similar to those in ischemic stroke, with slightly higher prevalence of hypertension in both AF and non‐AF individuals (Figures S13 and S14). The prevalence of vascular disease and heart failure was markedly lower in non‐AF ICH compared with AF‐related ICH.

### Other Analysis

We applied a fixed 7‐year look‐back period to define incident stroke, ensuring equal look‐back across the study period. Using this definition, 3.96% of all “incident” AF‐related strokes represented recurrent events occurring more than 7 years after a previous stroke event and an additional 0.02% were third events.

We performed sensitivity analyses for ischemic stroke and ICH using a fixed 7‐year look‐back period for AF and restricting follow‐up to when all the used registers were available (2015–2021). The results showed that the overall proportion of AF‐related strokes was similar (28.1% versus 29.4% with unrestricted look‐back for AF). Incidence rates for both AF‐ and non‐AF‐ related ischemic stroke and ICH were similar to the main analyses, with only marginal changes in estimates and overlapping confidence intervals. Sex‐ and age‐adjusted regression analysis yielded results consistent with the main analysis, with stable trends for AF related ischemic stroke (IRR 0.99; 95% CI, 0.92–1.06) and AF‐related ICH (0.98; 95% CI, 0.93–1.05).

## DISCUSSION

In this nationwide study of incident AF‐related strokes in Norway during 2001–2021, we report four key findings: (1) The proportion of AF‐related strokes increased from 22.0% in 2001 to 31.8% in 2021; (2) In contrast to the declining trend in non‐AF ischemic strokes, AF‐related ischemic stroke incidence remained stable overall, and increased in younger individuals (15–64 years) with AF; (3) AF‐related ICH rates increased in older individuals (≥75 years) with AF and remained stable in younger age groups; (4) The majority (68.5%) of AF‐related ischemic strokes occurred in non‐anticoagulated individuals, whereas 62.5% of AF‐related ICH occurred in OAC users.

Comparisons with existing literature warrant caution due to variations in study designs, methodology, diagnostics, population composition, and the management of AF over time. Differences may also arise from variations in the definition of AF‐associated stroke, the length of lookback period, and the use of post‐stroke investigations (eg, Holter monitoring).

Previous studies report conflicting trends in AF‐related ischemic stroke. A UK study (2005–2016) found that AF‐related strokes declined, while ICH incidence increased before stabilizing during the last 5 years.^7^ In a US study on patients with AF, ischemic stroke rates increased during 2011–2015 but declined during 2016 to 2021, while ICH increased initially, before stabilizing during 2016–2021.^12^ In the Swedish AF population aged ≥70 years, ischemic stroke incidence was stable during 2001 to 2012, and thereafter declined until 2020.^13^ The UK‐based Oxford Vascular Study (2002–2017) found no reduction in incident AF‐related strokes after 2007, and the proportion of ischemic strokes associated with AF remained high (32.6%).^2^ In a Finnish study (2007–2018), Teppo et al examined stroke risk before initiation of OAC in patients with AF and report a decline in ischemic stroke rate from 36.7 to 27.6 per 1.000 patients‐years, mainly driven by older high‐risk women, whereas rates remained stable in patients with a CHA_2_DS_2_‐VA score of 1.^14^ Consistent with these findings, a systematic review demonstrated that temporal trends in stroke incidence in high‐income countries differ by age, showing less favorable patterns among younger compared with older populations.^15^

Men carry a higher burden of AF, whereas female sex associates with greater ischemic stroke risk.^16^ However, sex differences in stroke risk seem to have flattened in recent data, reflected in the decision to remove the sex‐category from the CHA_2_DS_2_‐VASc score.^17^, ^18^ We observed similar temporal patterns in stroke incidence for women and men, but men had consistently higher rates. Ischemic stroke incidence rates declined among women aged >75 years, but not significantly in men in the corresponding age groups. These findings suggest that historical sex disparities in AF management and stroke prevention may be diminishing, although the lack of decline in men highlights the need for improved stroke prevention in older men.

Increasing stroke incidence over time among younger non‐AF individuals has also been reported in other studies.^8^ A recent UK population‐based cohort study found declining trends in stroke incidence among individuals ≥55 years but increase in younger age groups.^19^ In the younger stroke patients, traditional vascular risk factors were common, yet population‐level changes in these risk factors did not explain the rise. Moreover, transient ischemic attack incidence also increased significantly in those <55 years in the Oxfordshire study, as well as in the United States and Denmark, suggesting that diagnostic shifts from transient ischemic attack to stroke are unlikely the explanation.^19^, ^20^, ^21^ A Danish study reported increasing incidence of ischemic stroke and transient ischemic attack among individuals 15 to 30 years of age, while incidence of ICH was stable.^20^

Some studies in the general population have reported decreasing incidence of ICH, including recent data from Norway^8^, ^22^ whereas others have reported more stable incidences in recent years.^23^ A Danish study reported stable ICH occurrence, but the proportion of OAC‐related ICH increased from 12% to 23% during 2010–2017.^24^ Similarly, a French study reported an increase in AF‐related ICH from 17% in 2010 to 26% in 2017.^25^ In our study, the incidence of non‐AF‐related ICH decreased, whereas AF‐related ICH increased in older age groups. AF‐related ICH was stable in younger patients with AF and decreased in non‐anticoagulated patients with AF. These findings likely reflect increased OAC use in older patients with AF and underscores the importance of managing elevated bleeding risk in older patients through systematic reassessment of OAC indication, concomitant antiplatelet use, and management of frailty, risk of falls and other modifiable risk factors.

We observed an increasing trend of AF‐related SAH. Traumatic SAH (*ICD‐10* S06.6) was not included, and given that most SAH are aneurysmal, a causal link to rising OAC use seems unlikely. A more plausible explanation is improved AF ascertainment, shifting cases from non‐AF into the AF group. Previous studies have reported contradictory findings regarding an association between OAC and SAH and were limited by low power and unmeasured confounders.^26^, ^27^, ^28^, ^29^

Risk factors for stroke and AF are overlapping. Illicit drug use is more common in younger adults and is strongly associated with stroke.^30^ Other contributing factors include physical inactivity and increasing prevalence of obesity and diabetes. Recent studies from the U.S. and Europe report increased incidence of ischemic stroke in younger adults, accompanied by increasing prevalence of traditional risk factors, such as hypertension, dyslipidemia, diabetes, smoking and obesity.^30^, ^31^, ^32^ The stroke risk associated with AF increases with age, therefore prevention strategies focus mainly on older individuals. Early‐onset AF and its impact on stroke risk is understudied, although a recent U.S. study reported that younger age at AF diagnosis predicts earlier stroke, with a linear relationship between age at diagnosis and stroke risk.^33^ The Dutch YOUNG‐AF study found that 90% of patients with young‐onset AF had risk factors and 25% had familial AF; only 11% had no risk factors.^34^

We found an increase in the use of OAC before ischemic stroke in high‐risk AF patients, but a stable trend in patients with a CHA_2_DS_2_‐VA score of 1. In younger patients with AF and a low CHA_2_DS_2_‐VA score, the net clinical benefit of OAC use is not well established. Current guidelines suggest considering OAC in patients with AF with a CHA_2_DS_2_‐VA score of 1, while for patients with a CHA_2_DS_2_‐VA score of 2, OAC is strongly recommended.^18^, ^35^ Our findings emphasize the need for more refined risk stratification tools to better identify younger patients with AF who are truly at elevated stroke risk.

Nevertheless, even among high‐risk AF patients, only 45.7% were anticoagulated before the ischemic stroke, which may suggest missed opportunities for earlier AF detection or for timely reassessment of the indication for anticoagulation. Following the 2010 European guidelines recommending OAC over Aspirin for stroke prevention in AF, and the subsequent availability of novel OACs since 2012, OAC use has markedly increased.^36^, ^37^ A Danish study on patients with AF with ischemic stroke and CHA_2_DS_2_‐VA score of ≥1, found that nearly two‐third did not receive OAC as recommended by guidelines, but pre‐stroke OAC use increased to 58.5% in 2017.^38^ Similarly, we found that in high‐risk patients with known AF, OAC use before ischemic stroke increased from 40% to 64% in 2021, meaning 1/3 were not anticoagulated despite previous AF diagnosis. Reasons for non‐anticoagulation despite known AF are uncertain and may include contraindications, temporary treatment discontinuation, poor adherence, or patient preference.

Our research group recently published a study demonstrating that patients with AF initially classified as low‐risk experience a rapid increase in stroke risk over time, highlighting the importance of dynamic reassessment.^39^ The lower prevalence of heart failure and vascular disease in non‐AF strokes highlights comorbidity profiles that warrant tailored prevention strategies.

The incidence of AF‐related ischemic stroke in patients with known AF has declined, particularly in older age groups, likely reflecting increased OAC use during the study period. Ischemic stroke presenting with AF has remained stable whereas the incidence of ischemic stroke with AF diagnosed within 6 months has increased, especially in younger individuals. The observed increase in AF‐related strokes may not reflect a true rise in incidence. Possible explanations include improved AF detection through advances in technology and wearables, prolonged cardiac monitoring and changes in coding practices. A decline in other stroke subtypes, such as small vessel disease, may also increase the relative share of AF‐related strokes. Demographic aging and improved survival after myocardial infarction and heart failure may further enlarge the population at risk for developing AF. Secular increases in major AF risk factors, including hypertension and diabetes, likely contribute, and the rising prevalence of obesity is expected to increase risk of both AF and stroke.^40^ Last, increased survival among patients with known AF may result in more strokes, particularly among the oldest age groups. Although improved AF detection may partly explain the findings, they also indicate that a substantial proportion of AF remains undetected before stroke and improved AF detection strategies may still have potential to reduce stroke burden. Furthermore, 68.5% of AF‐related ischemic strokes occurred in non‐anticoagulated patients, indicating significant scope for improved prevention strategies.

The main strength of this study is the completeness of the nationwide registry data with a long observation period, allowing reliable ascertainment of incident events with an extended look‐back, and virtually no loss to follow‐up in the setting of a free health care system combined with mandatory registries.

Certain limitations should be acknowledged. We report descriptive statistical associations, which do not imply causality. Changes in clinical practice, guidelines, imaging access, and AF surveillance may confound temporal trends in registry data. Data on BMI, smoking, and physical inactivity were unavailable. Registry‐based studies are susceptible to misclassification due to potential coding errors and temporal changes of coding practice. However, the validity of AF, stroke, and CHA_2_DS_2_‐VA risk factors, is reported to be high in Norwegian registries.^41^, ^42^, ^43^, ^44^, ^45^

Using an unrestricted look‐back period may lead to more strokes being classified as AF‐related in recent years, yet sensitivity analyses using a fixed 7‐year look‐back aligned with the main findings. Conversely, the fixed 7‐year look‐back period for incident stroke may misclassify some recurrent strokes as incident events, but this approach avoids unequal look‐back windows that could bias incidence estimates over time, particularly in later periods. Nevertheless, only 4% of AF‐related strokes were recurrent, and a recent study from our group showed that such misclassification errors are stable, suggesting minimal impact on temporal trends.^8^

We report annual proportion of comorbidities, formal tests of trends were outside the scope of this study, and the findings do not imply any associations. The increased level of comorbidity may partly be explained by enhanced screening for risk factors and accumulation of diagnoses, particularly at older age. The thresholds for diagnosing hypertension and diabetes have also changed over the study period.

The study population was predominantly White patients, limiting generalizability to other populations.

Our findings have important clinical implications. The increasing incidence of AF‐related ischemic stroke in younger individuals suggests potential for improved AF detection before stroke. The increase in ICH in the oldest age group may partly reflect increased OAC use, and careful risk–benefit assessment of OAC and mitigation of bleeding risk in elderly patients remains essential. Furthermore, the substantial proportion of individuals with known AF not receiving OAC at the time of stroke highlights opportunities for improved prevention through systematic reassessment of OAC indication and review of treatment adherence.

## CONCLUSIONS

In this nationwide Norwegian registry study, the incidence of non‐AF‐related stroke declined over two decades. However, this favorable trend does not extend to AF‐related strokes. Ischemic stroke attributed to AF have increased among younger individuals, suggesting that AF has become accountable for a higher proportion of incident strokes or that AF detection has improved. Incidence of intracerebral hemorrhage have remained stable in the younger AF population, whereas the increase in the oldest age group may reflect changing patterns of OAC use. These findings underscore the need for continued efforts in early AF detection and optimized stroke prevention across all age groups.

## Sources of Funding

This study is funded by South‐Eastern Norway Regional Health Authority (26903) and the Research Council of Norway (336283).

## Disclosures

M.M. has received lecture fees from AstraZeneca, Bayer, Boehringer‐Ingelheim, Bristol Myers Squibb, MSD, and Pfizer. T.B. has received lecture fees from Bayer, Boehringer Ingelheim, Bristol Myers Squibb, and Pfizer. The disclosures are outside the submitted work.

## Supporting information

Tables S1–S7
Data S1–S3
Figures S1–S14
