# Supplementary material for: Trends in Incidence of Atrial Fibrillation–Related Stroke: The Norwegian AFNOR Study
Source: J Am Heart Assoc. 2026 Sep 9;15(19):e049696. doi: 10.1161/JAHA.126.049696 (PMC13745160; doi:10.1161/JAHA.126.049696)
Supplement: Supplementary file 1 [file JAH3-15-e049696-s001.pdf]

Supplemental Material

Supplemental Methods

Data S1. Definitions of incident stroke based on ICD-9 and ICD-10 codes

Outcome	ICD-9 codes	ICD-10 codes	Notes / Exclusion criteria
**Total stroke**	430<br>431<br>433<br>434<br>436	I60, I61, I63 (except I63.6), I64	No prior hospitalization for ischemic stroke, intracerebral hemorrhage, or unspecified stroke in the past 7 years. Stroke as secondary diagnosis excluded if primary diagnosis = previous stroke (ICD-9: 438; ICD-10: I69) or rehabilitation (ICD-9: V57; ICD-10: Z50.80, Z50.89).
**Ischemic stroke**	433<br>434	I63 (except I63.6)	No prior hospitalization for ischemic or unspecified stroke in the past 7 years. Same exclusion criteria as above.
**Intracerebral hemorrhage**	431	I61	No prior hospitalization for intracerebral hemorrhage in the past 7 years. Same exclusion criteria as above.
**Subarachnoid haemorrhage**	430	I60	No prior hospitalization for subarachnoid haemorrhage in the past 7 years. Same exclusion criteria as above.
**Unspecified stroke**	436	I64	No prior hospitalization for ischemic or unspecified stroke in the past 7 years. Same exclusion criteria as above.
**Transient ischemic attack (TIA)**	435	G45	No prior hospitalization for ischemic stroke, unspecified stroke, or TIA in the past 7 years. TIA as secondary diagnosis excluded if primary diagnosis = previous stroke (ICD-9: 438; ICD-10: I69) or rehabilitation (ICD-9: V57; ICD-10: Z50.80, Z50.89).

ICD10 = International Classification of Diseases, 10th revision. ICPC2 = International Classification of Primary Care, 2nd Edition.

Data S2: Definition of AF

	**Norwegian Patient Registry/ Historic hospitalizations**	**Norwegian Prescription Database**			**Control and Payment of Health Reimbursements Database**
	**ICD10 codes ICD9 codes**	**ATC codes**	**Reimbursement codes**		**ICPC2 codes**
			**ICD10 codes**	**ICPC2 codes**	
Atrial fibrillation	ICD10: I48<br>ICD9: 427.3	B01AA03<br>B01AE07<br>B01AF01<br>B01AF02	I48	K78	K78

Data S3. Definition of the CHA_2DS_2-VA score

Comorbidity	Norwegian Patient Registry	Norwegian Prescription Database			Control and Payment of Health Reimbursements Database
	ICD10 codes	ATC codes	Reimbursement codes		ICPC2 codes
			ICD10 codes	ICPC2 codes	
Congestive heart failure	I11.0 I42.0 I50 I13.0 I13.2	C01AA04 C01AA05 C01DA08 C01EB17 C02DB02 C03AA01 C03AA03 C03AB01 C03CA01 C03CA02 C03DA01 C03DA02 C03DA04 C03EA01 C07AA05 C07AB02 C07AB07 C07AG02 C09AA01 C09AA02 C09AA03 C09AA05 C09AA10 C09CA01 C09CA03 C09CA06 R03DA04	I11.0 I42 I50 I50.1 J81	K77	K77
Hypertension	I10 I11 I12 I13 I15 O10 O11	C02A C02C C02DB C02DC C03A C03E C07A C07B C08 C09 C03CA01 C03CA04 C03DA01 C03DB01 (÷ C07AA07) (÷ C08CA06)	I10 I11 I12 I13 I15 O10 O11	K86 K87	K86 K87
Diabetes mellitus	E10 E11 E12 E13 E14 O24.0 O24.1 O24.2 O24.3	A10			T89 T90
Ischemic stroke, transient ischemic attack, arterial thromboembolism	I63 I64 I74 G45.8 G45.9 G45	B01AB04 B01AB05 B01AC04 B01AC05 B01AC07 B01AC30	G45 I63 I74	K89 K90	K89 K90
Vascular disease	I21 I22 I70 I73.9 I.23 I.71 I.72 I25.2	B01AB01 B01AB04 B01AB05 B01AC04 B01AC07 B01AC22 B01AC24 B01AC26 C01DA02 C01DA08 C01DA14 C04AD03 C07AA05 C07AB02 C07AB03 C07AB07 C07AG01 C07AG02 C08CA01 C08CA02 C08CA05 C08DA01 C08DB01 C09AA01 C09AA03 C09AA05 C10	-22 I21 I22 I70 I73.9	-22 K75 K92	K75 K92

An incident event of **total stroke** was defined as an inpatient hospitalization with a main or secondary diagnosis of stroke, or out-of-hospital death with stroke as the underlying cause. Total stroke included ischemic stroke, intracerebral hemorrhage, subarachnoid haemorrhage and unspecified stroke. Cases were included only if no hospitalization for any stroke subtype had occurred in the previous seven years. Stroke as secondary diagnosis was not included if the primary diagnosis was previous stroke (ICD-9: 438; ICD-10: I69) or rehabilitation (ICD-9: V57; ICD-10: Z50.80, Z50.89).

Ischemic stroke was defined as hospitalization with a main or secondary diagnosis of ischemic stroke with no previous hospitalization for ischemic or unspecified stroke in the past seven years or out of hospital death with ischemic stroke as the underlying cause. Exclusions followed the same rules as for total stroke.

Intracerebral hemorrhage was defined as hospitalization with a main or secondary diagnosis of intracerebral hemorrhage or out hospital death with ICH as the underlying cause with no previous hospitalization for ICH in the past seven years. Exclusions followed the same rules as for total stroke.

Unspecified stroke was defined as hospitalization or out of hospital death with a main or secondary diagnosis of unspecified stroke with no previous hospitalization for ischemic or unspecified stroke in the past seven years. Exclusions followed the same rules as for total stroke.

Transient ischemic attack (TIA) was defined as hospitalization with a main or secondary diagnosis of TIA with no previous hospitalization for ischemic stroke, unspecified stroke, or TIA in the past seven years. TIA as secondary diagnosis was not included if the primary diagnosis was previous stroke (ICD-9: 438; ICD-10: I69) or rehabilitation (ICD-9: V57; ICD-10: Z50.80, Z50.89).

Subarachnoid haemorrhage (SAH) was defined as hospitalization or out of hospital death with main or secondary diagnosis of SAH and no previous hospitalization with SAH during the past seven years. We only included non-traumatic SAH.

Patients were identified using national health care registers, including hospitalizations and outpatient specialist visits from the Norwegian Patient Registry (NPR), the Norwegian Cause of Death Registry, reimbursements in the Norwegian Prescription Database (NorPD), historical cardiovascular disease hospitalization database at the University of Bergen, and primary health care (KUHR). The NPR and historical cardiovascular disease database provided primary and secondary hospital discharge diagnoses according to the International Classification of Diseases (ICD), 9th and 10th edition. There are no private health institutions with emergency treatment of stroke in Norway, therefore these two data sources cover all treated strokes from 1994 onwards.

<u>Data sources have the following period of data availability:</u> the Norwegian Patient Registry (since 2008), the Hospital Research (FS) Database at University of Bergen (1994 -2009), Norwegian Control and Payment of Health Reimbursements Database since 2006), the Norwegian Prescription Database (from 2004), the Cause of Death Registry (since 1951); and Statistics Norway (since 1960).

Supplementary Tables

Table S1. Stroke cases and proportions of AF-related and non-AF-related total stroke

Year	Cases N	AF cases	Non-AF cases	Proportion AF%	Proportion Non AF%
2001	11772	2592	9180,00	22,0	78,0
2002	12103	2784	9319,00	23,0	77,0
2003	12074	2846	9228,00	23,6	76,4
2004	11597	2815	8782,00	24,3	75,7
2005	11516	3074	8442,00	26,7	73,3
2006	11592	3121	8471,00	26,9	73,1
2007	11247	3153	8094,00	28,0	72,0
2008	11494	3442	8052,00	29,9	70,1
2009	10848	3463	7385,00	31,9	68,1
2010	10991	3267	7724,00	29,7	70,3
2011	10880	3329	7551,00	30,6	69,4
2012	10807	3344	7463,00	30,9	69,1
2013	10226	3261	6965,00	31,9	68,1
2014	10179	3248	6931,00	31,9	68,1
2015	9786	3120	6666,00	31,9	68,1
2016	10157	3435	6722,00	33,8	66,2
2017	10024	3297	6727,00	32,9	67,1
2018	9894	3283	6611,00	33,2	66,8
2019	10158	3314	6844,00	32,6	67,4
2020	9969	3345	6624,00	33,6	66,4
2021	10261	3262	6999,00	31,8	68,2

Table S2. Cases and proportions of stroke subtypes

	All strokes		AF		Non-AF	
**Stroke subtype**	**Cases N**	**Proportion**	**Cases N**	**Proportion (%)**	**Cases N**	**Proportion (%)**
**ICH**	29,932	13.15	7,503	11.23	22,429	13.95
**IS**	163,008	71.63	51,059	76.44	111,949	69.63
**SAH**	8,644	3.80	1,051	1.57	7,593	4.72
**US**	25,991	11.42	7,182	10.75	18,809	11.70

Table S3. Incidence rate ratios (IRR) from 2001 to 2021 for AF-related and non-AF-related ischemic stroke

	AF		Non-AF	
	**IRR (95% CI)**	**p-value**	**IRR (95% CI)**	**p-value**
**Both sexes:**				
**All**	1,01 (1,00–1,03)	0,165	0,98 (0,97-0,99)	<0,001
**15–44**	1,07 (1,04–1,09)	<0,001	1,00 (0,99-1,00)	0,189
**45–64**	1,03 (1,02–1,04)	<0,001	0,98 (0,98-0,99)	<0,001
**65-74**	1,00 (1,00–1,01)	0,072	0,97 (0,97-0,98)	<0,001
**75–84**	0,99 (0,99–0,99)	<0,001	0,97 (0,96-0,97)	<0,001
**85+**	1,00 (0,99–1,00)	0,314	0,97 (0,97-0,97)	<0,001
**Men:**				
**All**	1,01 (0,99–1,03)	0,378	0,98 (0,96-0,99)	0,003
**15–44**	1,06 (1,02–1,09)	0,001	1,00 (0,99-1,00)	0,247
**45–64**	1,02 (1,01–1,03)	<0,001	0,99 (0,98-0,99)	<0,001
**65-74**	1,00 (1,00–1,01)	0,117	0,97 (0,97-0,98)	<0,001

**75–84**	1,00 (0,99–1,00)	0,263	0,96 (0,96-0,97)	<0,001
**85+**	1,00 (1,00–1,01)	0,251	0,97 (0,96-0,97)	<0,001
**Women:**				
**All**	1,01 (0,99–1,03)	0,233	0,98 (0,97-0,99)	<0,001
**15–44**	1,09 (1,04–1,13)	<0,001	1,00 (0,99-1,01)	0,498
**45–64**	1,04 (1,03–1,05)	<0,001	0,98 (0,98-0,98)	<0,001
**65-74**	1,00 (1,00–1,01)	0,362	0,98 (0,97-0,98)	<0,001
**75–84**	0,99 (0,98–0,99)	<0,001	0,97 (0,96-0,97)	<0,001
**85+**	0,99 (0,99–1,00)	0,021	0,98 (0,97-0,98)	<0,001

Adjusted for sex and age. IRR; incidence rate ratio.

Table S4. Incidence rate ratios (IRR) from 2001 to 2021 for AF-related and non-AF-related intracerebral haemorrhage (ICH).

	**AF**		**Non-AF**	
	**IRR (95% CI)**	**p-value**	**IRR (95% CI)**	**p-value**
**Both sexes:**				
**All**	1,01 (1,00-1,02)	0,191	0,98 (0,97-0,99)	<0,001
**15–44**	1,02 (0,95-1,09)	0,607	1,00 (0,99-1,01)	0,437
**45–64**	1,00 (0,99-1,02)	0,652	0,98 (0,98-0,99)	<0,001
**65-74**	0,99 (0,98-1,00)	0,065	0,97 (0,97-0,98)	<0,001
**75–84**	1,02 (1,01-1,03)	<0,001	0,97 (0,97-0,98)	<0,001
**85+**	1,04 (1,03-1,04)	<0,001	0,98 (0,98-0,99)	<0,001
**Men:**				
**All**	1,01 (0,99-1,03)	0,529	0,98 (0,97-0,99)	<0,001
**15–44**	1,02 (0,94-1,11)	0,595	1,00 (0,99-1,01)	0,667
**45–64**	1,00 (0,98-1,03)	0,846	0,98 (0,98-0,99)	<0,001
**65-74**	0,99 (0,98-1,00)	0,066	0,97 (0,96-0,97)	<0,001
**75–84**	1,02 (1,01-1,03)	0,004	0,97 (0,96-0,98)	<0,001
**85+**	1,04 (1,02-1,06)	<0,001	0,97 (0,97-0,98)	<0,001
**Women:**				
**All**	1,01 (1,00-1,03)	0,157	0,98 (0,97-0,99)	0,001
**15–44**	1,01 (0,87-1,16)	0,917	0,99 (0,98-1,01)	0,446
**45–64**	1,01 (0,98-1,04)	0,574	0,98 (0,98-0,99)	<0,001
**65-74**	1,00 (0,98-1,01)	0,567	0,98 (0,97-0,99)	<0,001
**75–84**	1,02 (1,01-1,03)	0,001	0,97 (0,97-0,98)	<0,001
**85+**	1,03 (1,02-1,04)	<0,001	0,99 (0,98-1,00)	0,002

Adjusted for sex and age. IRR, incidence rate ratio.

Table S5. IRR from 2001 to 2021 in AF and non-AF related subarachnoid haemorrhage (SAH) stratified by age, adjusted for sex and age.

	**AF**		**Non-AF**	
	**IRR (95% CI)**	**p-value**	**IRR (95% CI)**	**p-value**
**All**	1,04 (1,03-1,05)	<0,001	0,98 (0,97-0,99)	<0,001
**15–44**	1,05 (0,94-1,19)	0,387	0,96 (0,95-0,97)	<0,001
**45–64**	1,03 (1,00-1,06)	0,035	0,98 (0,97-0,98)	<0,001
**65-74**	1,04 (1,01-1,06)	0,004	0,99 (0,98-1,00)	0,009
**75–84**	1,03 (1,01-1,05)	<0,001	0,99 (0,98-1,00)	0,110
**85+**	1,06 (1,04-1,08)	<0,001	0,99 (0,98-1,00)	0,121

Adjusted for sex and age. IRR, incidence rate ratio.

Table S6. Incidence rate ratios (IRR) from 2001 to 2021 for AF-related and non-AF-related total stroke

	**AF**		**Non-AF**	
	**IRR (95% CI)**	**p-value**	**IRR (95% CI)**	**p-value**
Both sexes				
**All**	1,00 (0,99-1,02)	0,324	0,97 (0,96-0,98)	<0,001
**15–44**	1,06 (1,03-1,09)	<0,001	0,99 (0,99-0,99)	<0,001
**45–64**	1,02 (1,02-1,03)	<0,001	0,98 (0,98-0,98)	<0,001
**65-74**	1,00 (1,00-1,00)	0,646	0,97 (0,97-0,97)	<0,001
**75–84**	0,99 (0,99-0,99)	<0,001	0,96 (0,96-0,96)	<0,001
**85+**	1,00 (0,99-1,00)	0,318	0,96 (0,96-0,96)	<0,001
Menn				
**All**	1,00 (0,98-1,03)	0,562	0,97 (0,96-0,99)	<0,001
**15–44**	1,05 (1,02-1,09)	0,001	0,99 (0,99-0,99)	0,001
**45–64**	1,02 (1,01-1,02)	<0,001	0,98 (0,98-0,98)	<0,001
**65-74**	1,00 (0,99-1,00)	0,718	0,97 (0,96-0,97)	<0,001
**75–84**	1,00 (0,99-1,00)	0,217	0,96 (0,95-0,96)	<0,001
**85+**	1,00 (0,99-1,00)	0,494	0,96 (0,95-0,96)	<0,001
Women				
**All**	1,01 (0,99-1,03)	0,356	0,97 (0,96-0,98)	<0,001
**15–44**	1,08 (1,04-1,12)	<0,001	0,99 (0,98-0,99)	0,001
**45–64**	1,04 (1,02-1,05)	<0,001	0,98 (0,97-0,98)	<0,001
**65-74**	1,00 (0,99-1,01)	0,776	0,97 (0,97-0,98)	<0,001
**75–84**	0,99 (0,98-0,99)	<0,001	0,96 (0,96-0,97)	<0,001
**85+**	0,99 (0,99-1,00)	0,050	0,97 (0,96-0,97)	<0,001

Adjusted for sex and age. IRR, incidence rate ratio.

Table S7. Proportion of AF patients using oral anticoagulation (OAC) before ischemic stroke, by CHA_2DS_2-VA stratum

**Year**	**All**	**CHA_2DS_2-VA score 0 or 1**	**CHA_2DS_2-VA score =1**	**CHA_2DS_2-VA score ≥2**
2008	26,32	17,41	15,97	27,07
2009	25,55	10,30	13,71	26,60
2010	29,64	13,61	14,41	30,85
2011	28,89	9,78	9,02	31,37
2012	32,81	12,57	12,57	34,30
2013	30,86	4,76	5,74	32,73
2014	30,62	9,96	10,43	32,76
2015	32,79	12,90	15,87	34,46
2016	33,75	10,80	12,53	35,78
2017	34,20	8,47	11,89	37,01
2018	38,14	9,13	11,03	40,96
2019	39,25	10,43	12,69	41,76
2020	39,67	9,24	12,65	42,71
2021	42,57	8,97	10,88	45,71

**Proportion of AF patients using OAC before ischemic stroke in each stratum of CHA_2DS_2-VA score. Values are row percentages: numerator = AF patients in the given CHA_2DS_2-VA stratum who were on OAC before the index stroke; denominator = all AF patients with ischemic stroke in the same stratum. The estimates in 2013 should be interpreted with caution, as the dip in OAC use this year likely reflects changes in prescription recording during the transition to electronic prescribing in 2013, rather than a true change in prescription pattern.*

Figure S1. Incidence rates of AF-related ischemic strokes, stratified by sex and age

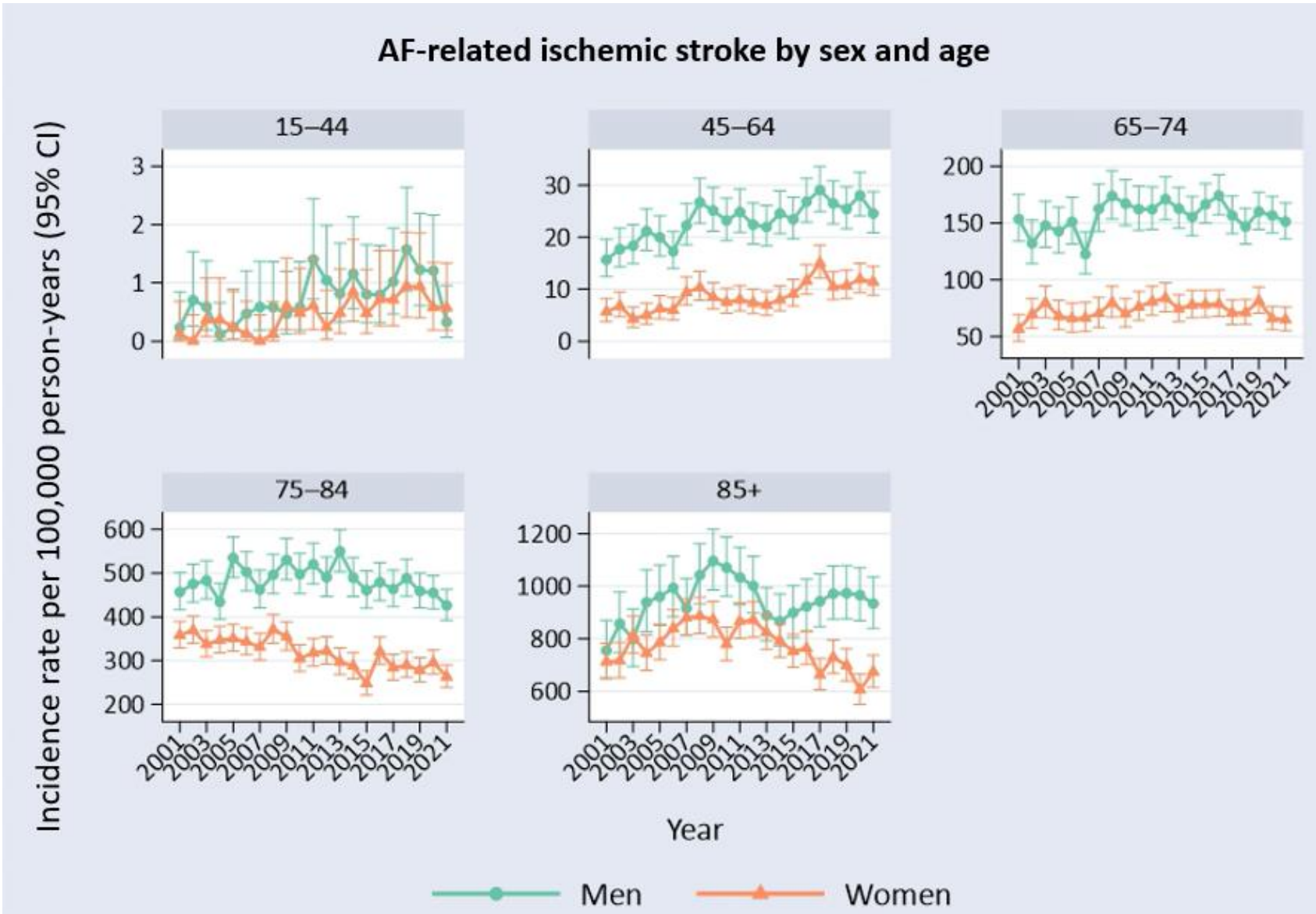

Y-axis scales differ between panels. AF; Atrial Fibrillation, CI; Confidence Interval

Figure S2. Incidence rates of AF-related ischemic stroke, stratified by timing of AF diagnosis

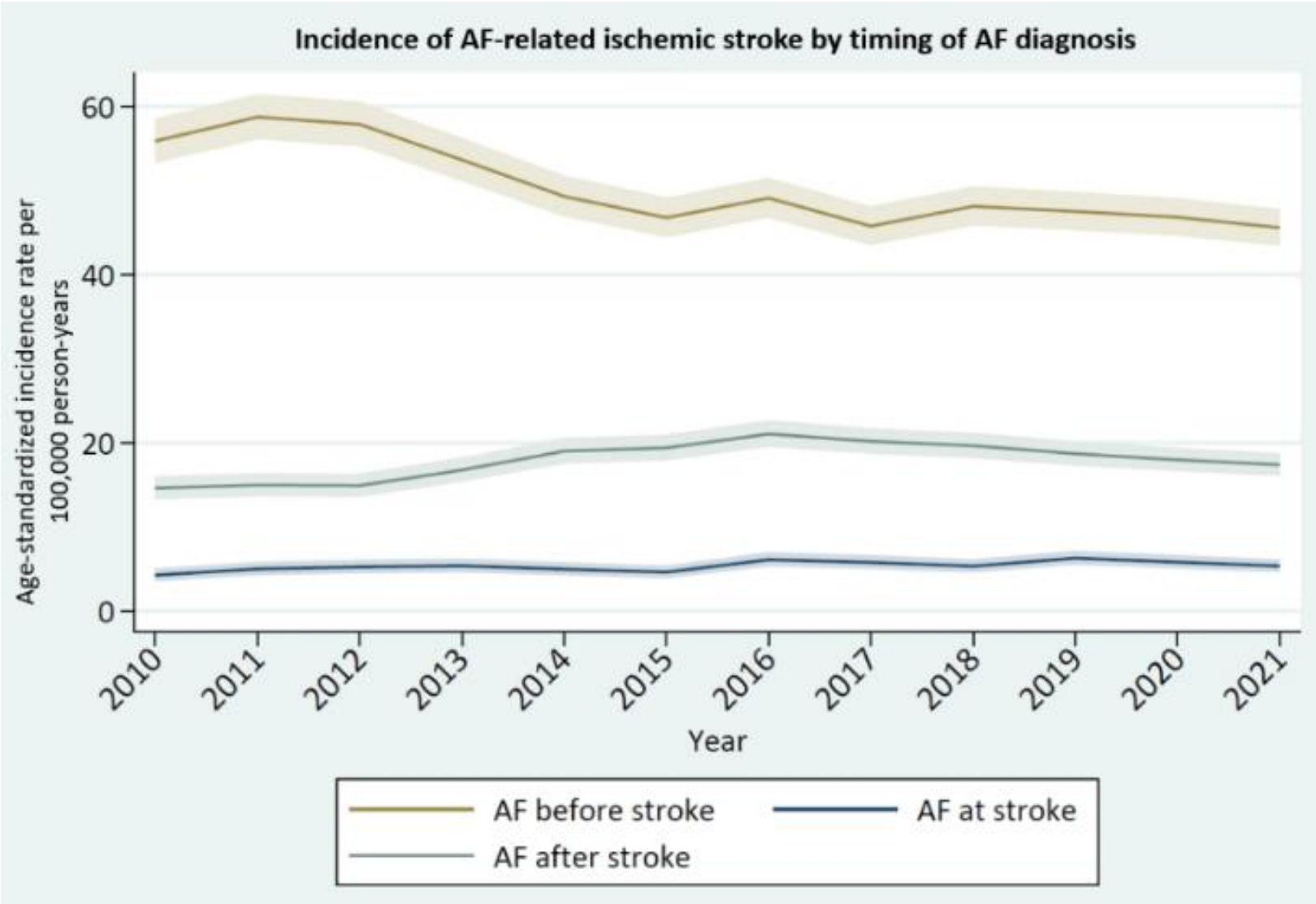

Annual incidence rate of ischemic stroke in patients with known AF (AF registered before stroke date), AF diagnosed at stroke date and AF diagnosed within 6 months after stroke date. AF; Atrial Fibrillation

Figure S3. Incidence rates of AF-related ischemic stroke, stratified by timing of AF diagnosis and age

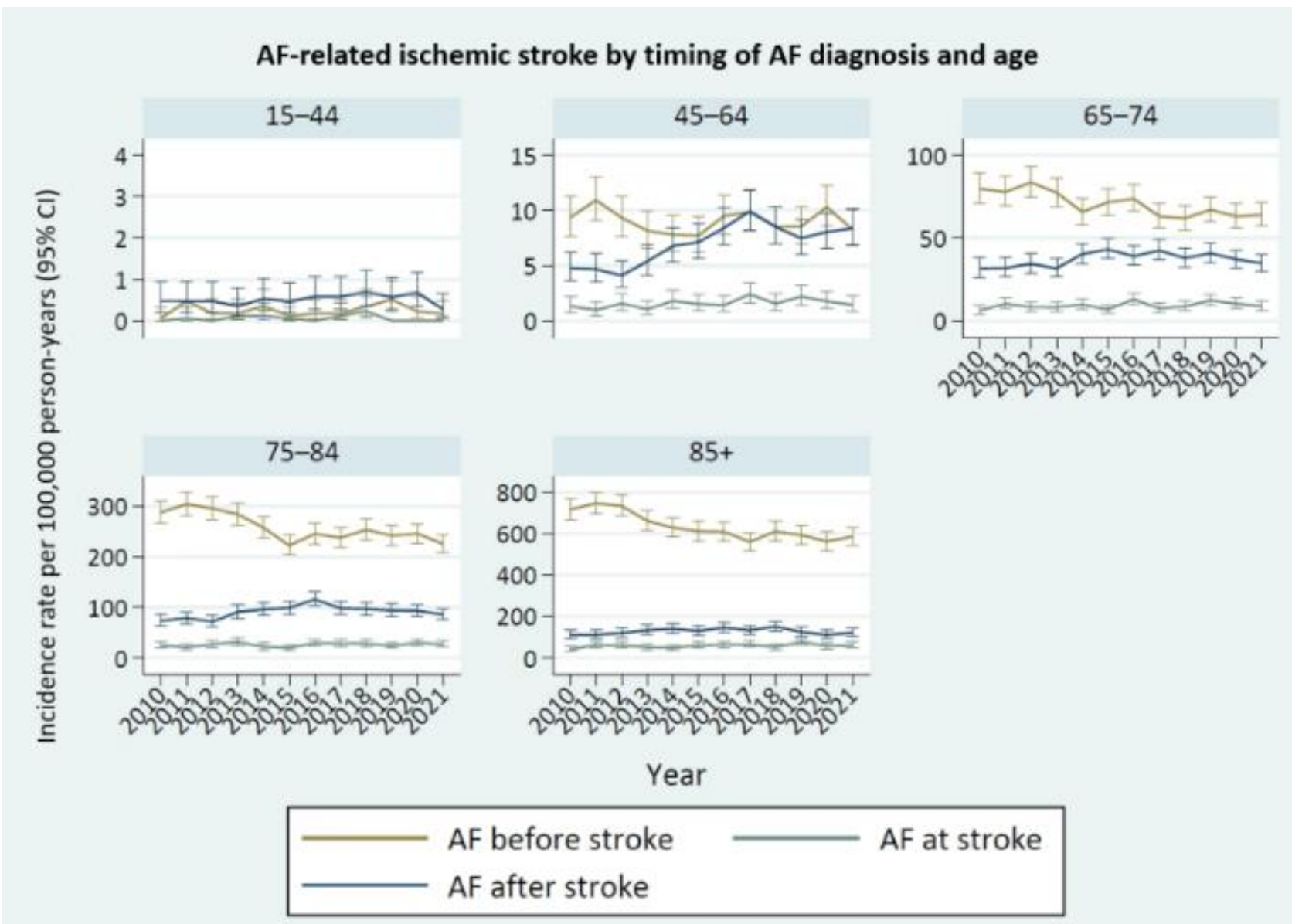

Y-axis scales differ between panels. AF; Atrial Fibrillation, CI; Confidence Interval

Figure S4. Incidence rates of AF-related intracerebral haemorrhage, stratified by OAC use

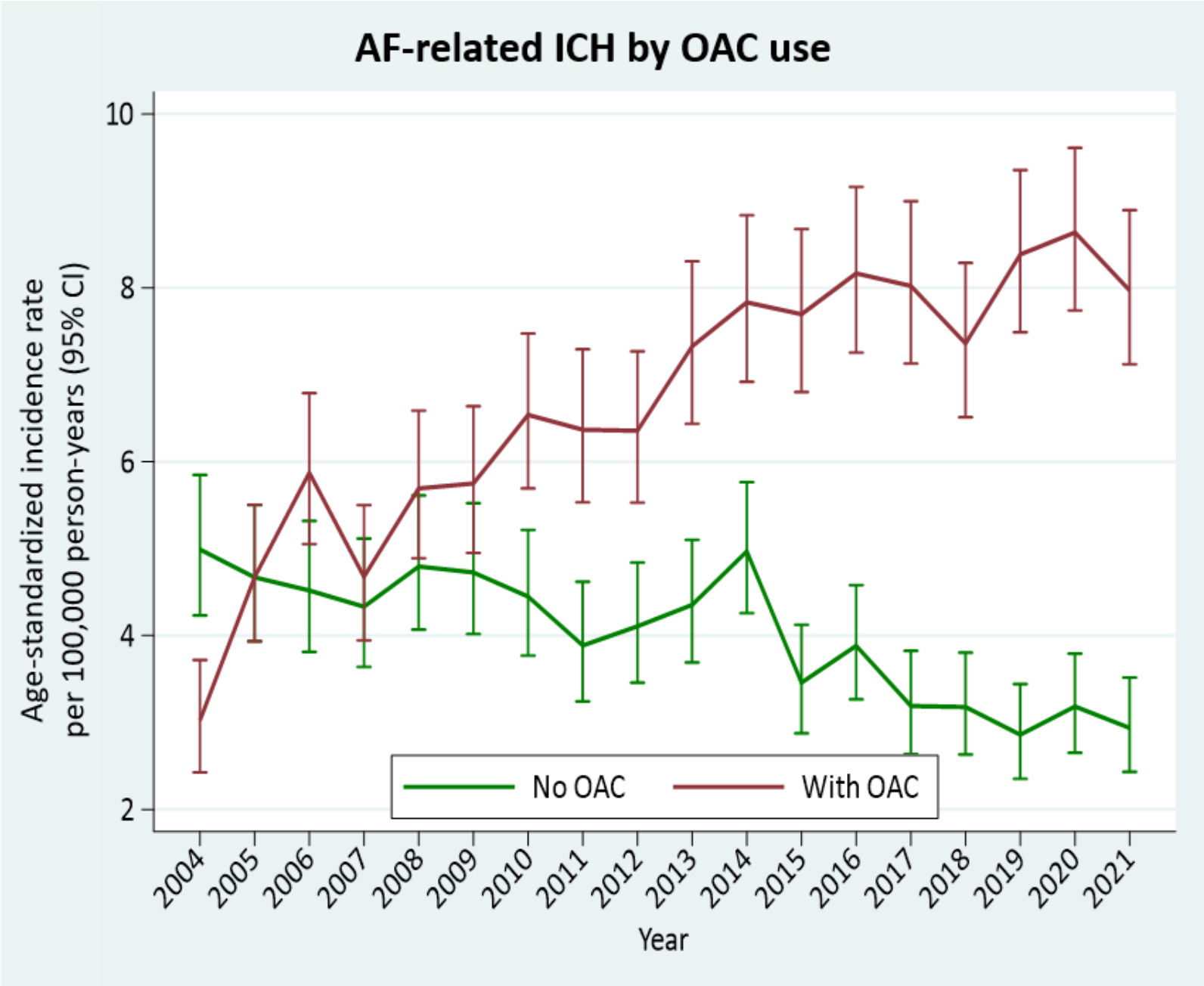

AF; Atrial Fibrillation, ICH; intracerebral haemorrhage, OAC; oral anticoagulation

Figure S5. Incidence rates of AF-related and non-AF-related subarachnoid haemorrhage

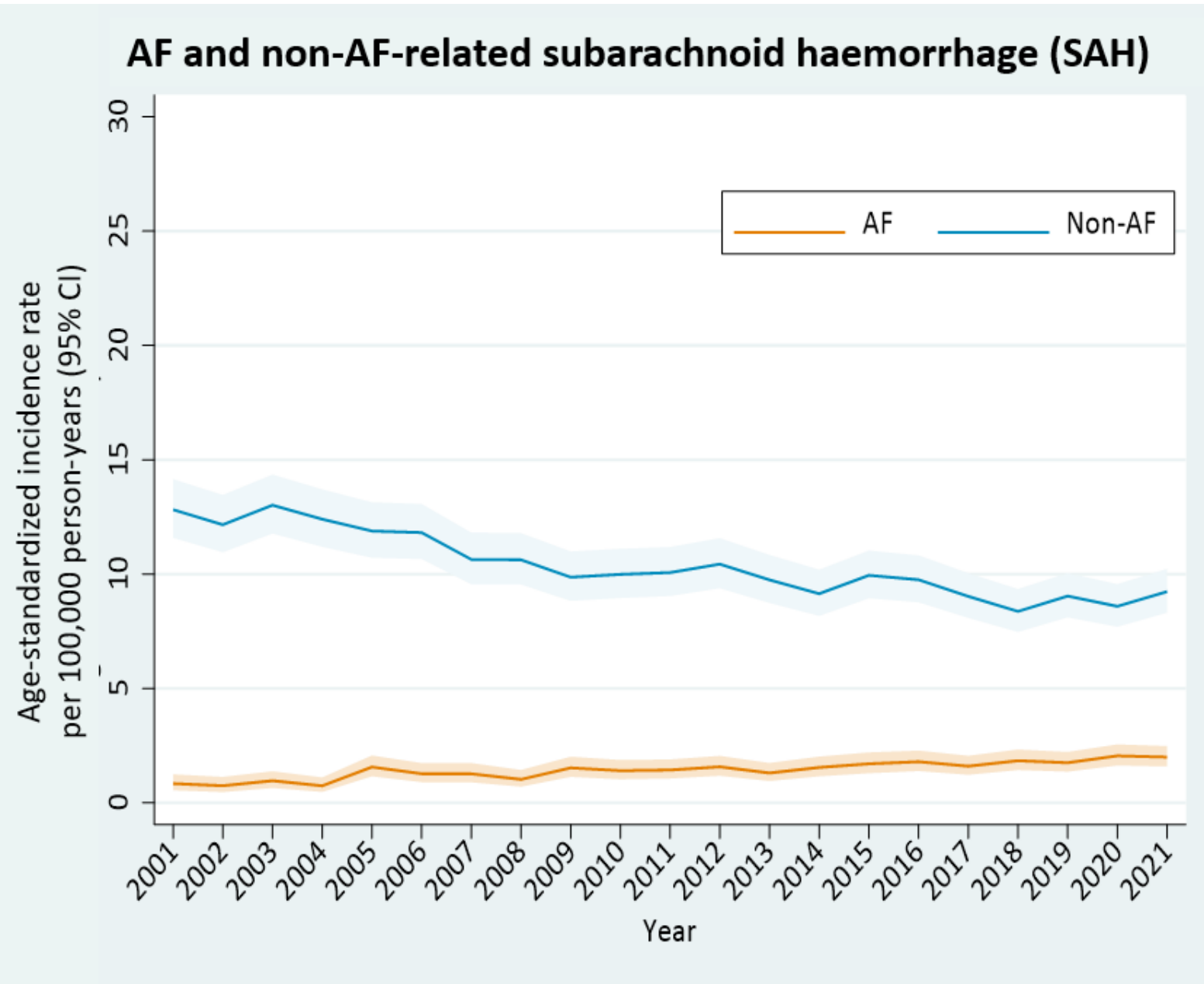

AF; Atrial Fibrillation, SAH; subarachnoid haemorrhage

Figure S6. Incidence rates of AF-related and non-AF-related unspecified stroke, stratified by sex

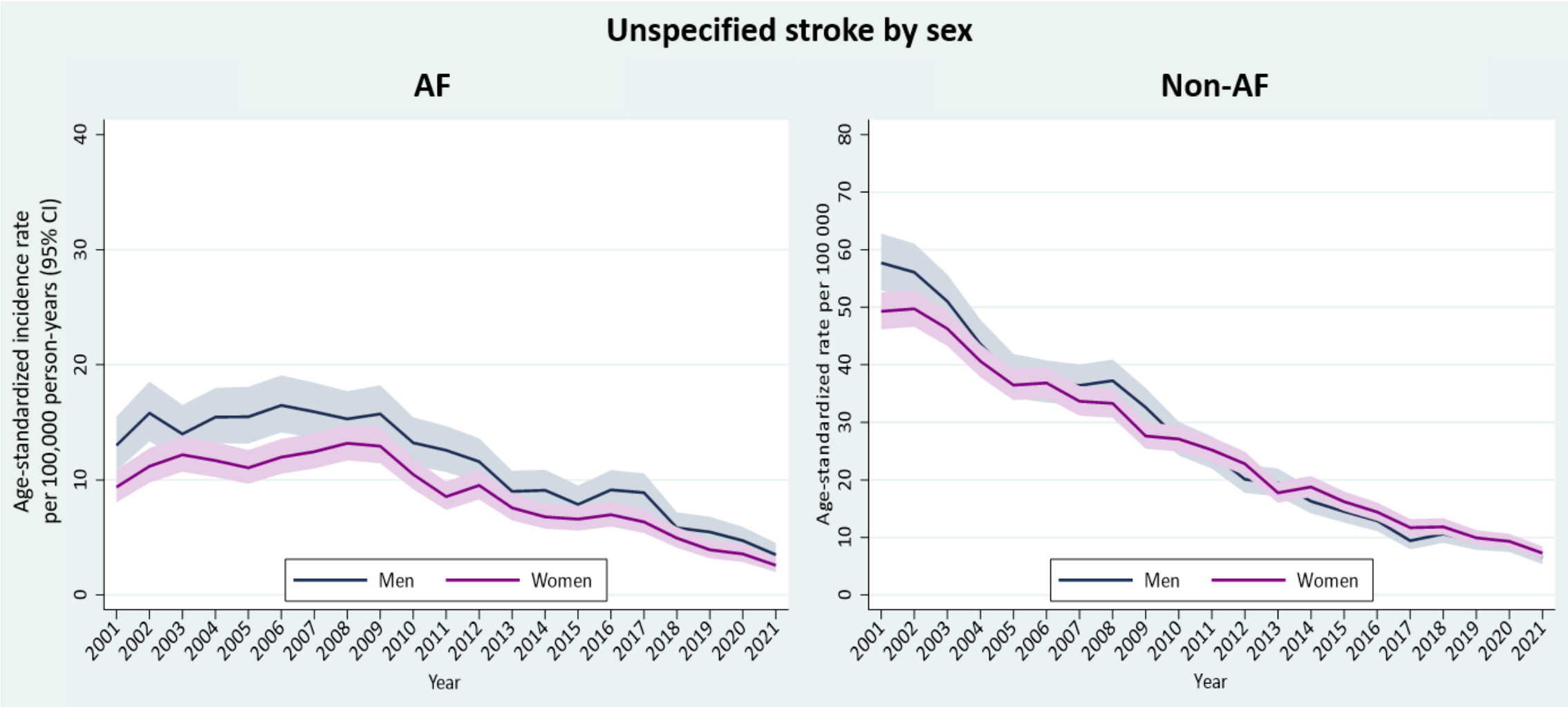

AF; Atrial Fibrillation, CI; Confidence Interval

Figure S7. Incidence rates of AF-related and non-AF-related total stroke, stratified by sex

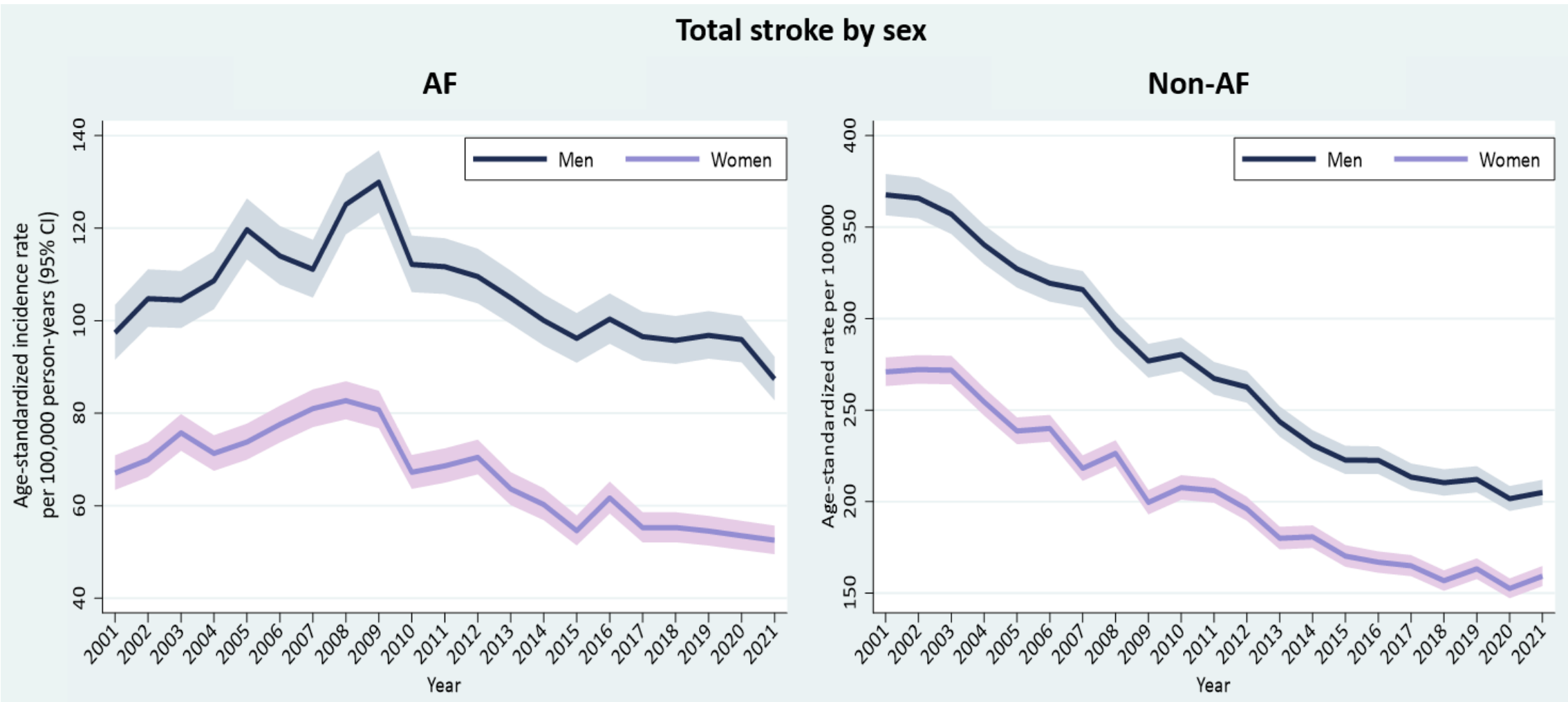

AF; Atrial Fibrillation, CI; Confidence Interval

Figure S8. Incidence rates of AF-related and non-AF related total stroke, stratified by age

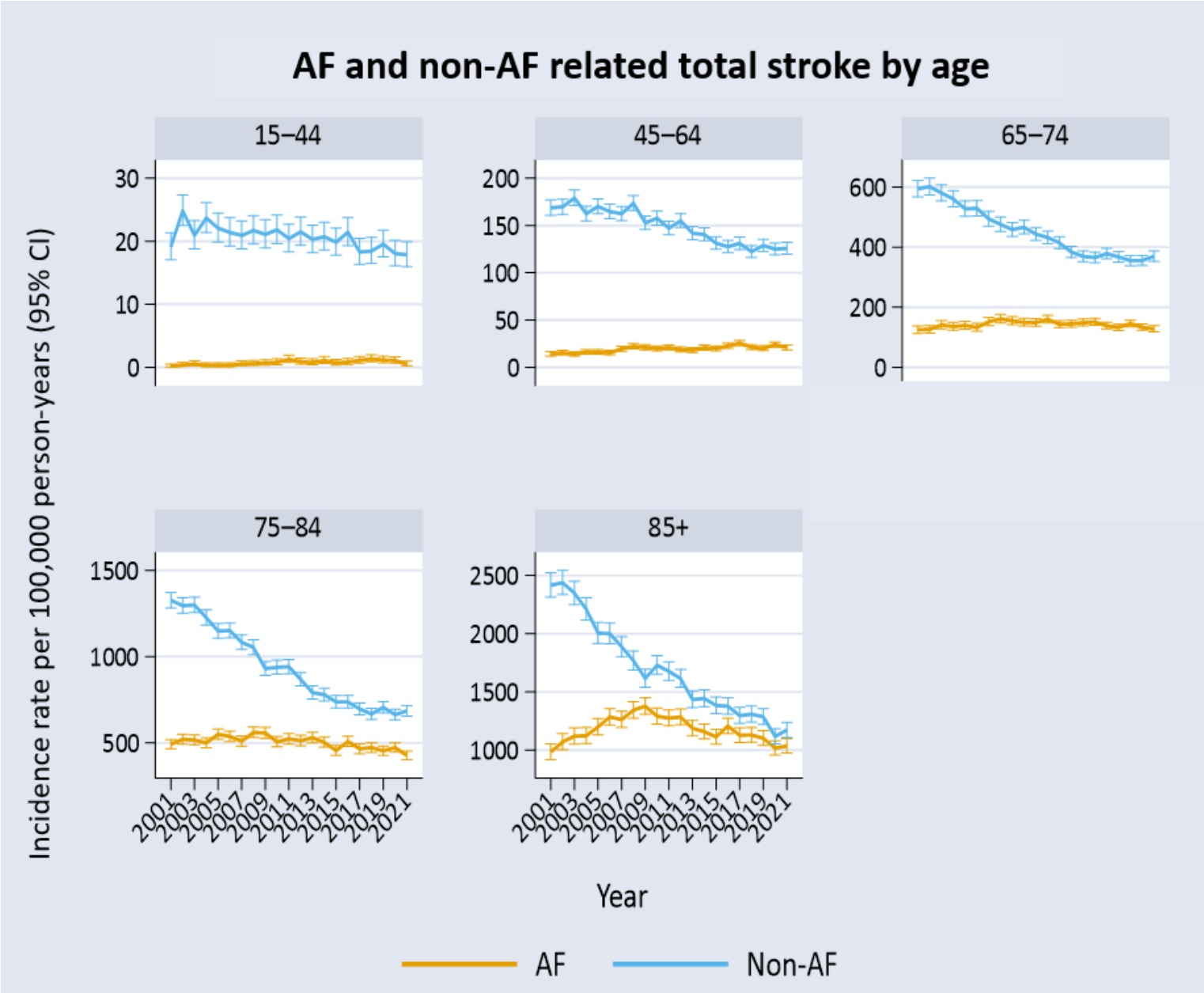

AF; Atrial Fibrillation, CI; Confidence Interval

Figure S9. Incidence rate of AF-related and non-AF related total stroke, stratified by age and sex

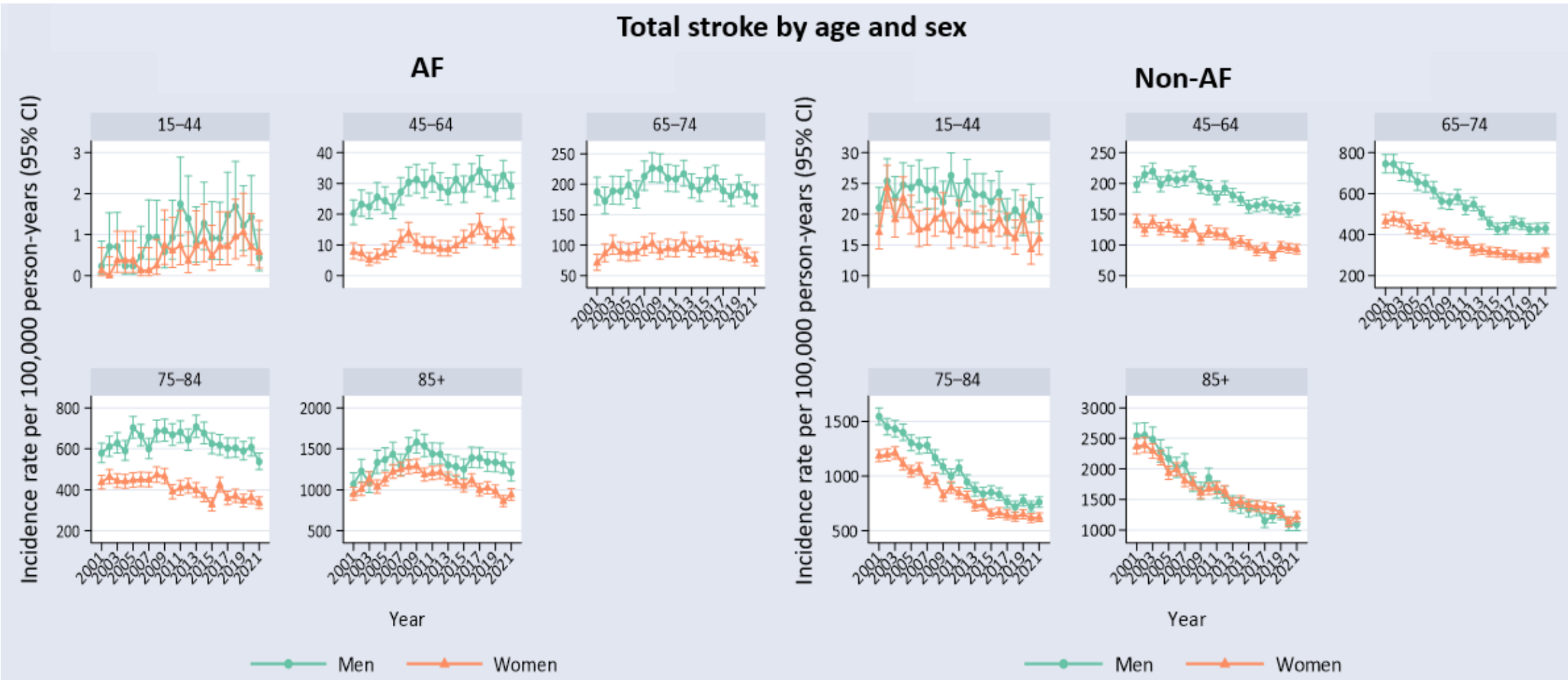

AF; Atrial Fibrillation, CI; Confidence Interval

Figure S10. Annual proportion of oral anticoagulant users with AF-related ischemic stroke and ICH, stratified by stroke sex and age

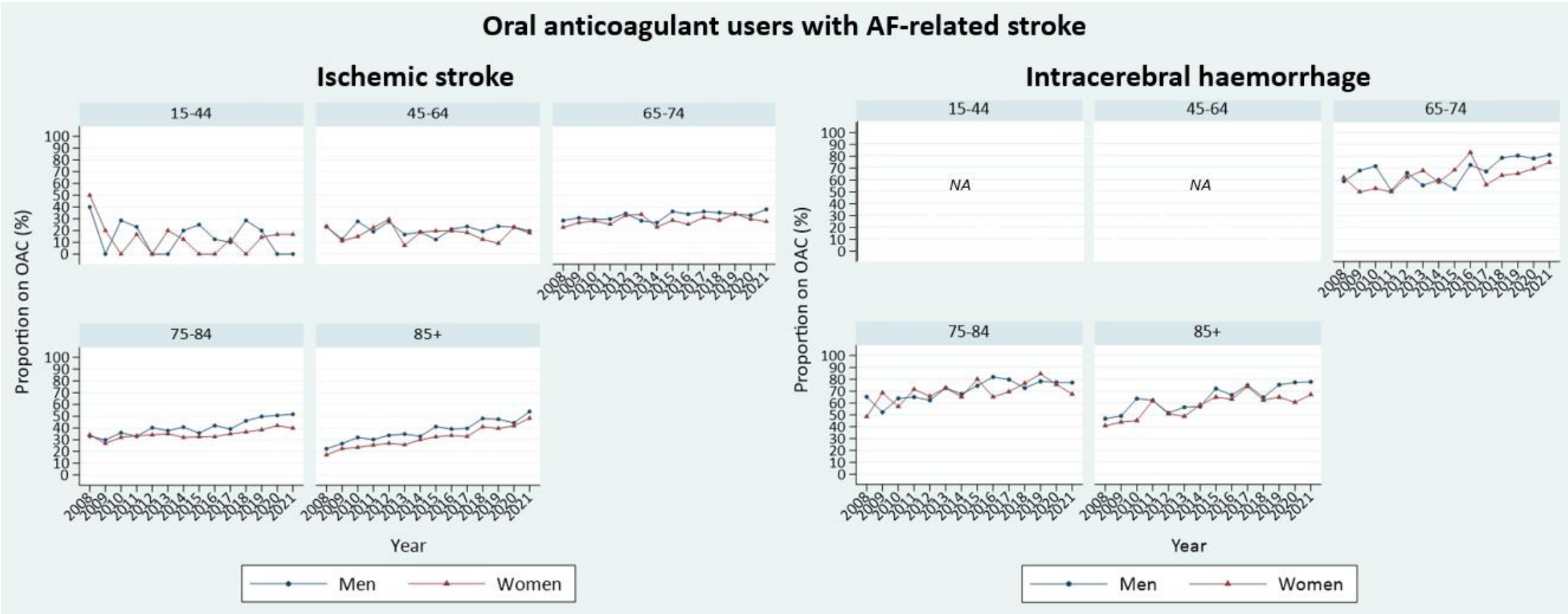

Results for ICH in age group 15-44 and 45-54 were not applicable. AF; Atrial Fibrillation, ICH; intracerebral haemorrhage, NA; not applicable

Figure S11. Annual proportion of OAC users in AF-related ischemic stroke with known AF diagnosis prior to stroke, stratified by CHA_2DS_2-VA score

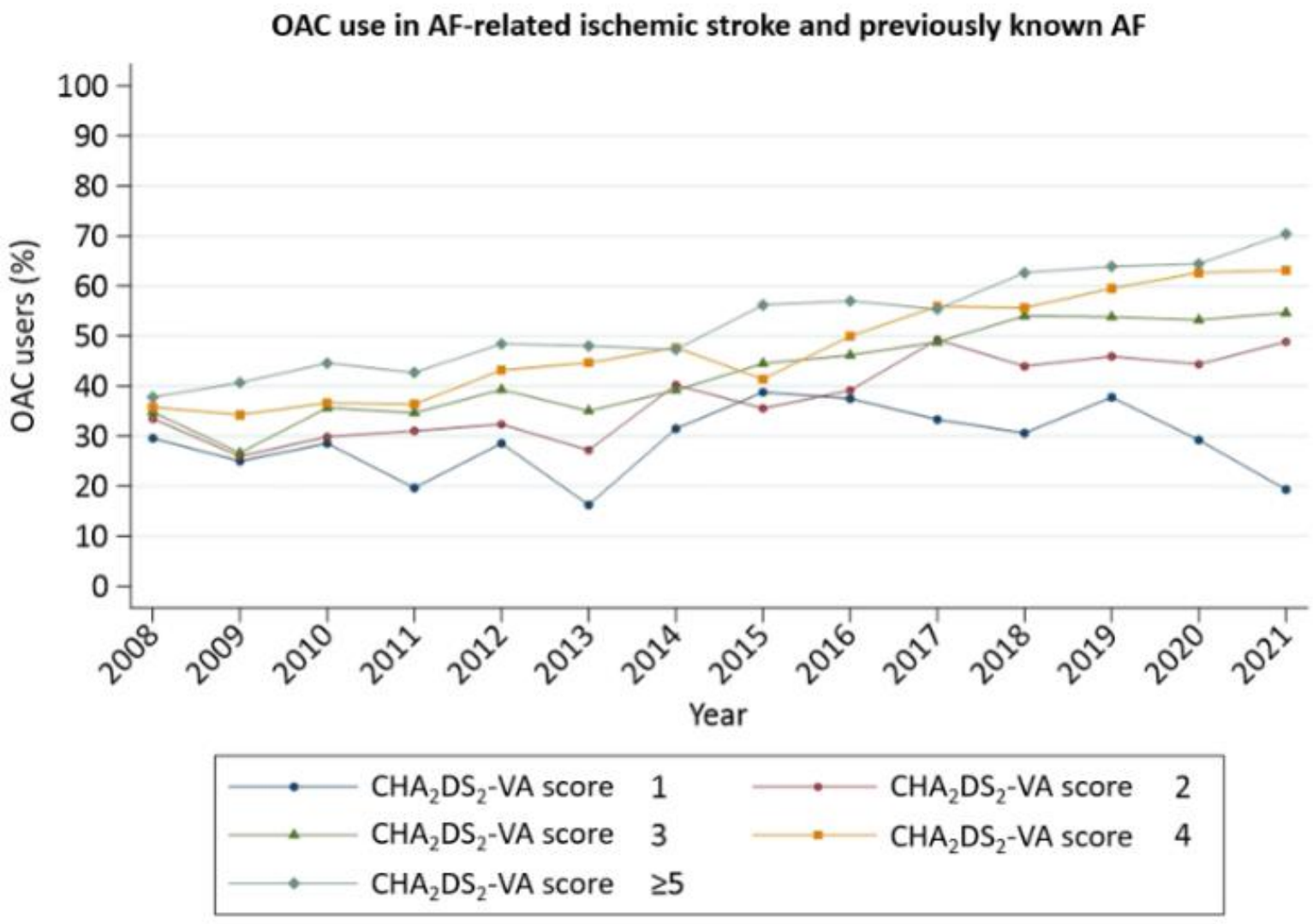

The figure shows the annual proportion of individual receiving OAC (%) at time of stroke among those with AF-related ischemic stroke and previously known AF (i.e. AF registered prior to stroke), stratified by the CHA_2DS_2-VA score. AF; Atrial Fibrillation, OAC; Oral Anticoagulation.

Figure S12. Annual proportions of comorbidities among patients with AF-related and non-AF-related ischemic stroke, stratified by age

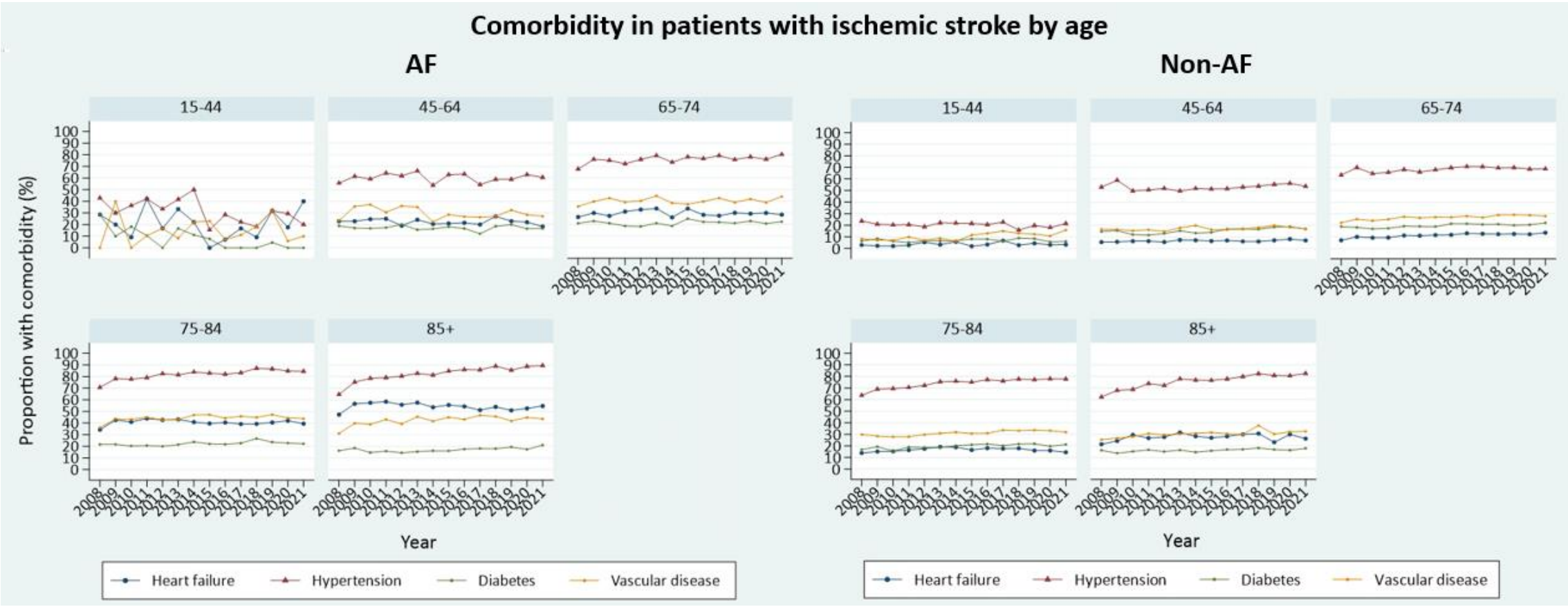

The small sample size in the youngest age group (15-44 years) limits precision, and results should be interpreted cautiously.

Figure S13. Annual proportions of comorbidities among patients with AF-related and non-AF-related intracerebral haemorrhage

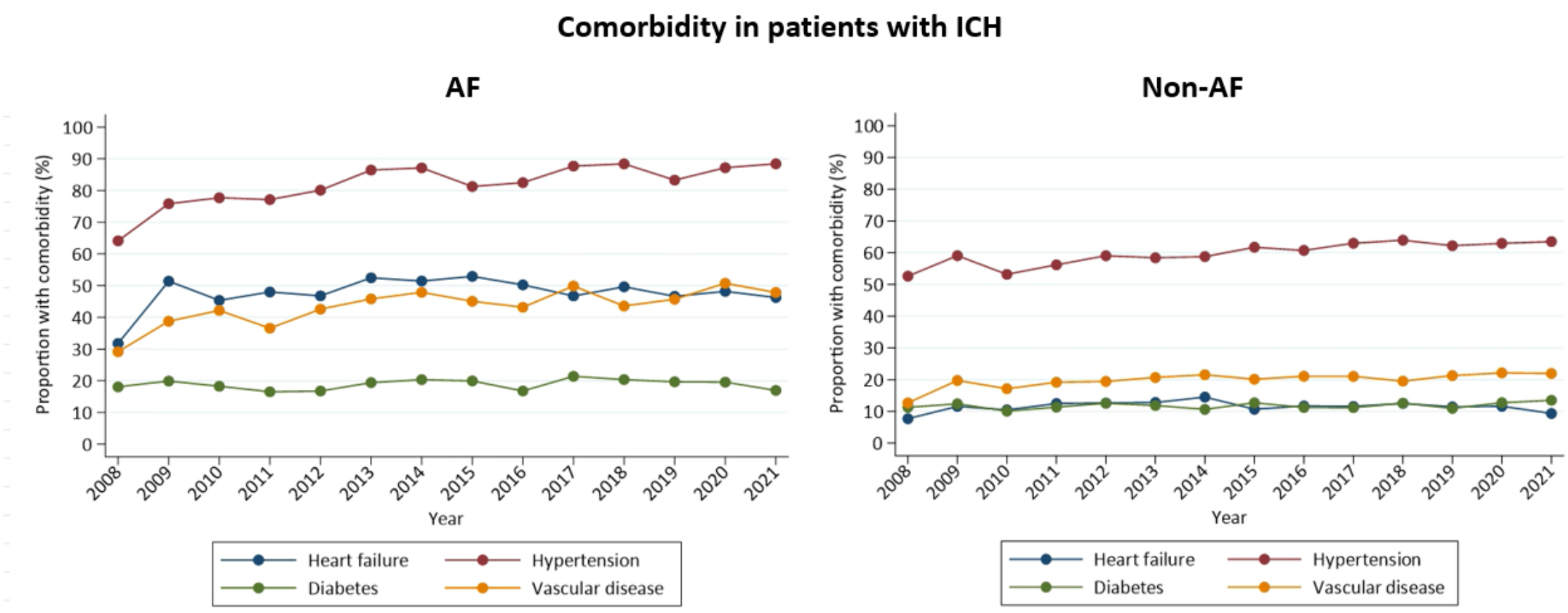

AF; Atrial Fibrillation, ICH; intracerebral haemorrhage

Figure S14. Annual proportion of comorbidities among patients with AF-related and non-AF-related intracerebral haemorrhage, stratified by age

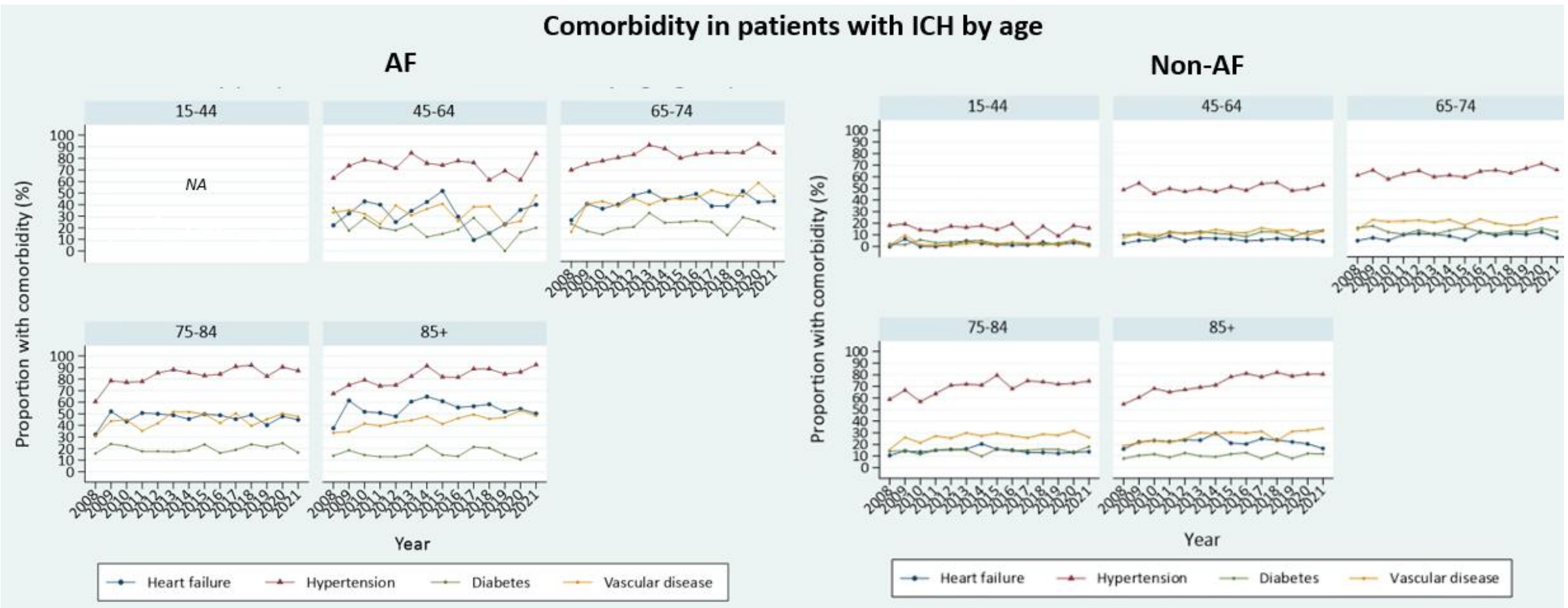

Results for age group 15-44 were not applicable. AF; Atrial Fibrillation, ICH; intracerebral haemorrhage
